# Effect of health warning labels on motivation towards energy-dense snack foods: Two experimental studies

**DOI:** 10.1016/j.appet.2022.106084

**Published:** 2022-08-01

**Authors:** Minna Ventsel, Emily Pechey, Katie De-loyde, Mark A. Pilling, Richard W. Morris, Giulia Maistrello, Hisham Ziauddeen, Theresa M. Marteau, Gareth J. Hollands, Paul C. Fletcher

**Affiliations:** a Behaviour and Health Research Unit, Department of Public Health and Primary Care, University of Cambridge, Forvie Site, Robinson Way, CB2 0SR, Cambridge, UK; b Tobacco and Alcohol Research Group, School of Psychological Science, University of Bristol, 12a Priory Road, BS8 1TU, Bristol, UK; c Bristol Medical School, University of Bristol, 5 Tyndall Avenue, BS8 1UD, Bristol, UK; d Department of Psychiatry, University of Cambridge, Douglas House, 18b Trumpington Rd, CB2 8AH, Cambridge, UK; e Cambridgeshire and Peterborough NHS Foundation Trust, Elizabeth House, CB21 5EF, Cambridge, UK; f Wellcome Trust–MRC Institute of Metabolic Science — Metabolic Research Laboratories, Addenbrooke's Hospital, CB2 0QQ, Cambridge, UK; g EPPI-Centre, UCL Social Research Institute, University College London, London, UK

**Keywords:** Choice behavior, Implicit motivation, Explicit motivation, Approach-avoidance, Snack selection, Manikin task

## Abstract

Health warning labels (HWLs) show promise in reducing motivation towards energy-dense snack foods. Understanding the underlying mechanisms could optimise their effectiveness. In two experimental studies in general population samples (Study 1 n = 90; Study 2 n = 1382), we compared the effects of HWLs and irrelevant aversive labels (IALs) on implicit (approach) motivation towards unhealthy snacks, using an approach-avoidance task (Study 1), and a manikin task (Study 2). We also assessed explicit motivation towards unhealthy snacks using food selection tasks. We examined whether labelling effects on motivation arose from the creation of outcome-dependent associations between the food and its health consequences or from simple, non-specific aversive associations. Both label types reduced motivation towards snack foods but only when the label was physically present. HWLs and IALs showed similar effects on implicit motivation, although HWLs reduced explicit motivation more than IALs. Thus, aversive HWLs appear to act both through low level associative mechanisms affecting implicit motivation, and by additionally emphasizing explicit causal links to health outcomes thereby affecting explicitly motivated choice behaviours.

## 1 Introduction

Excess consumption of energy-dense foods is a key driver of rising levels of obesity, itself a risk factor for a range of non-communicable and, latterly, communicable diseases (GBD 2015 Obesity Collaborators, 2017; Roberto et al., 2015; Swinburn et al., 2011). Labelling the energy or nutritional content of foods is one possible intervention that offers a scalable and potentially cost-effective means of modifying choice, purchase and consumption behaviours (Crockett et al., 2018; Shangguan et al., 2019). However, mere provision of information appears to have limited effects when implemented in real-world settings (Vasiljevic et al., 2018, 2019). An alternative approach is the use of labels that include aversive images depicting the negative consequences of consuming energy-dense foods. There is good evidence for the contribution of such health warning labels (HWLs) to smoking cessation, with a greater effect of image-and-text HWLs (also known as ‘pictorial’ or ‘graphic’) than text-only HWLs (Brewer et al., 2016; Hammond, 2011; Noar, Hall, Brewer, Monárrez-Espino, & Galanti, 2015). There is a smaller body of evidence on effects of HWLs applied to food and alcohol products (Clarke et al., 2021b; Grummon & Hall, 2020). Currently, specific evidence regarding the effectiveness of HWLs in reducing selection and consumption of foods high in fat, sugar and salt is limited, but preliminary research finds image-and-text HWLs increasing dietary self-control in relation to snack foods (Rosenblatt, Bode, et al., 2018; Rosenblatt, Summerell, et al., 2018), and reducing hypothetical selection of energy-dense snacks (Clarke et al., 2020).

While initial findings are promising, a clearer understanding of the underlying mechanisms by which HWLs exert their effects on selection and consumption could help optimise the design and delivery of such interventions. These mechanisms remain largely unknown, though a few studies have focused on the effects of aversive labelling on explicit and implicit attitudes or on dietary self-control (*e.g.* Asbridge, Pechey, Marteau, & Hollands, 2021; Hollands & Marteau, 2016; Hollands, Prestwich, & Marteau, 2011; Rosenblatt, Summerell, et al., 2018). One possibility is that they act by eliciting negative emotional arousal. This is supported by the observation that HWLs that elicit high negative emotional arousal are effective at reducing selection of alcoholic drinks (Clarke et al., 2021c), sugar sweetened beverages (Grummon & Brewer, 2020; Grummon et al., 2019; Mantzari, Vasiljevic, Turney, Pilling, & Marteau, 2018), and energy-dense snacks (Clarke et al., 2020). Negative emotional arousal is also a proposed mediator of the effects of HWLs on selection behaviour (Grummon & Brewer, 2020; Mantzari et al., 2018). This is consistent with tobacco research, where the increase in quit attempts produced by image-and-text HWLs is thought to be driven by their ability to elicit negative emotions, namely fear, disgust, discomfort and worry (Brewer et al., 2016, 2019; Cho et al., 2018). However, an important question is whether the arousal is elicited by the generally aversive visual nature of the HWL or by the fact that it emphasises a specific causal link between the behaviour and the aversive consequences. Are the effects of labelling driven primarily by a simple aversive association, or is it necessary for the label to depict a causal relationship between the product and the unpleasant outcomes?

In the current two experiments, we manipulated the nature of the aversive label in order to dissociate these mechanisms. Specifically, if a simple stimulus-response effect underlies the effect of labelling then an aversive label that depicts an outcome that is not causally relevant to the targeted behaviour should be equally effective in reducing that behaviour. Conversely, if the effect draws on goal-directed learning, then the causal relationship between the HWL and the behaviour may be key to maximizing the impact. That is, a HWL describing the unpleasant consequences of type 2 diabetes would exert a greater effect on responses to a chocolate bar than an aversive label unrelated to the effects of such consumption. In two studies, we compared aversive labels depicting an outcome that was causally related to over-consumption of such snacks (HWLs) and labels depicting an equally aversive stimulus that was unrelated to this behaviour (irrelevant aversive labels; IALs). For Study 1, we hypothesised that exposure to aversive labels (i) reduces implicit motivation (approach behaviour) for energy-dense snacks, with reductions being greater for HWLs than IALs; and (ii) reduces explicit motivation (liking, wanting, selection) for energy-dense snacks, with reductions being greater for HWLs than IALs. In Study 2, we hypothesised that HWLs presented at the time of implicit motivation measurement (rather than during a prior conditioning phase as in Study 1) affects approach behaviour and subsequent explicit motivation measures, again to a greater degree than IALs. We also hypothesised that the effect of exposure to aversive labels is greater in reducing energy-dense snack selection when products in the selection task display aversive labels, compared to when they are unlabelled.

### 1.1 Study 1 – Laboratory study of the effects of associative conditioning with aversive labels on subsequent motivation towards chocolate bars

We aimed to determine the effects of HWLs, IALs and no label on implicit motivation (approach/avoid behaviour) and explicit motivation (wanting, liking and selection) towards energy-dense foods with high reward value – chocolate bars. The primary aim was to estimate the impact of exposure to aversive labels (both HWLs and IALs) on implicit motivation towards energy-dense snack foods (chocolate bars), and to determine whether the nature of the aversive label would have an impact on any motivational effects. Specifically, we wished to understand if the effectiveness of the label required that the aversive label depicted an outcome causally related to the unhealthy snack or whether such effects might also be provoked by any aversive label irrespective of its causal relevance to unhealthy snack consumption.

## 2 Methods

The study protocol was preregistered on the Open Science Framework (OSF), prior to data collection (https://osf.io/pc4t6). Ethical approval for this study was granted by the Cambridge Psychology Research Ethics Committee (reference: PRE.2019.015).

### 2.1 Design

Using a between-subjects design, participants were randomised to one of the three study groups:

1. HWL group: Participants were presented with chocolate bars with labels that contained aversive images and warning texts that were relevant to excess calorie consumption.
2. IAL group: Participants were presented with chocolate bars with labels that contained aversive images and warning texts that were not relevant to excess calorie consumption.
3. No label control group: Participants were presented with chocolate bars with no labels.

The study had three stages: (i) pre-conditioning, when chocolate bars were initially presented unlabelled to obtain baseline measures of motivation; (ii) conditioning, when participants were presented with images of the chocolate bars displaying HWLs, IALs or no labels (depending on their assigned group) and required to perform an incidental task; (iii) post-conditioning, when chocolate bars were unlabelled.

### 2.2 Participants

The general population sample of 90 adult participants – comprising similar proportions of men and women and similar proportions of lower and higher socio-economic position – was recruited via a research agency. Participants were over 18 years old, liked and consumed chocolate at least once a week. Participants were fluent in English, had basic computer literacy (*i.e.* were able to use a computer for simple tasks), and able to provide written informed consent. All participants were asked to attend for testing on one occasion.

#### 2.2.1 Sample size determination

We were unaware of sufficiently similar previous studies that could meaningfully inform any power calculation. We were therefore guided primarily by practical considerations of available resources. 84 participants would allow detection of a large effect size (*d* = 0.7) of the manipulation on the primary outcome measure of implicit motivation, with alpha level of 5% and power of 80%. 90 participants were recruited to allow for some loss of data, and is consistent with convention (Lancaster, Dodd, & Williamson, 2004).

### 2.3 Materials

**Fig. 1:**
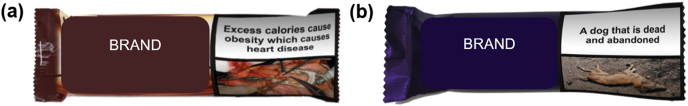
Examples of chocolate bars with (a) a HWL and (b) an IAL.

Four HWLs and four IALs were displayed on images of four well-known branded chocolate bars (Mars, Galaxy, Dairy Milk and Snickers). The labels were superimposed onto the chocolate bar packaging by a professional graphic designer, to represent what an actual labelled chocolate bar could look like. This was done while also ensuring visibility of both the label and the chocolate bar branding (Fig. 1). The HWLs depicted adverse health conditions that are directly or indirectly influenced by excess calorie consumption (such as obesity, cancer and cardiovascular disease), accompanied by brief explanatory text – used in previous studies on energy-dense snack labelling (Clarke et al., 2020). The IALs, selected via an internal pilot, comprised images unrelated to ill-health, such as dead, injured, or aggressive animals, with accompanying text statements unrelated to consumption of the labelled food. Full details on label development can be seen in the supplementary material (S1 and S2).

### 2.4 Measures

#### 2.4.1 Primary outcome measure

##### 2.4.1.1 Implicit motivation

A joystick (Thrustmaster T 16000M FCS) was used for the implicit motivation joystick task used to quantify speed of response to either an “approach” or an “avoid” instruction in relation to chocolate bar images, pre- and post-conditioning (each containing 48 trials [24 approach and 24 avoid]). The avoid instruction was indicated by an upward arrow, and prompted the participant to push the joystick away from themselves. The response resulted in shrinkage of the image (as though it were moving away). The approach instruction was indicated by a downward arrow, and prompted the participant to pull the joystick toward themselves. The response resulted in enlargement of the image (as though it were being pulled towards the participant) (Supplementary material S3). A slower reaction time when pushing the chocolate bar away from them, or a faster reaction time when pulling the chocolate bar towards them, would indicate an approach bias towards the chocolate. The implicit motivation measure was calculated as ‘avoid reaction time’ (a mean from 24 trials) minus ‘approach reaction time’ (a mean from 24 trials). This was done for both the pre-conditioning phase and the post-conditioning phase. Thus, a negative implicit motivation (a longer approach time than avoid time) indicates less motivation to approach the chocolate bar, and a positive implicit motivation time (a shorter approach time than avoid time) indicates more motivation to approach the chocolate bar.

#### 2.4.2 Secondary outcome measures

##### 2.4.2.1 Explicit liking and wanting

Assessed pre- and post-conditioning on 100 mm visual analogue scales, labelled at either end by ‘not at all’ (0), to ‘very’ (100), in response to two questions that were based on standard measures used in studies of food and drugs (Rogers & Hardman, 2015):

Liking: “How much do you like this chocolate bar generally?”
Wanting: “How much do you want one of these chocolate bars right now?”

##### 2.4.2.2 Snack selection

To investigate whether a participant selected an energy-dense chocolate bar or a healthier snack. Assessed post-conditioning, via an in-person snack selection task comprising eight snacks (the four energy-dense chocolate bars previously viewed, and four healthier alternatives) (Supplementary material S4). All the healthier snack options were 100 kcal or less per pack, while the energy-dense chocolate bars were all 200 kcal or more per pack. None of the snacks had labels on.

##### 2.4.2.3 Associative learning

The degree to which each chocolate bar became associated with the label it displayed, assessed post-conditioning. Assessed in HWL and IAL groups only, via a forced choice response post-conditioning in which participants had to recall which warning labels had been displayed on which chocolate bar. With a total of four chocolate bars and four labels to match, possible scores ranged from 0 (no correct pairings) to 4 (all correct pairings). This provided a measure of explicit learning of the associations.

#### 2.4.3 Additional measures

##### 2.4.3.1 Hunger

Assessed post-conditioning via a 100 mm visual analogue scale; “At the moment, how hungry are you?” (0; Not at all – 100; Very).

##### 2.4.3.2 Time since last ate

Assessed post-conditioning via a multiple choice question with 4 possible answers [Less than an hour ago/1–2 h ago/3–4 h ago/5 or more hours ago].

##### 2.4.3.3 Demographic characteristics

Assessed post-conditioning, including age, gender, ethnicity, highest education level, income level, height and weight.

### 2.5 Procedure

**Fig. 2:**
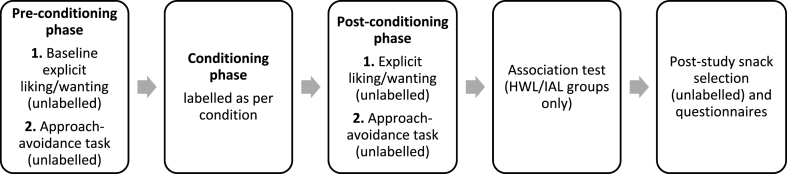
The flow of the steps of Study 1.

The flow of the procedure is illustrated in Fig. 2. Upon arrival at the laboratory, participants were given a cover story on the information sheet that the research was exploring thoughts and feelings towards different types of food. They then provided written informed consent to participate in the study and were informed that they could withdraw from the study at any time.

#### 2.5.1 Pre-conditioning phase

All participants firstly viewed images of the four unlabelled chocolate bars and rated explicit liking and wanting for each chocolate bar. They then completed the pre-conditioning approach-avoidance joystick task where unlabelled chocolate bars were displayed.

#### 2.5.2 Conditioning phase

Participants were randomised to the HWL, IAL or no label group. Randomisation was programmed into the task software (Matlab). During the conditioning phase each chocolate bar appeared on the screen at random intervals, paired with either a HWL, IAL or no label, as per the participants’ study group. This occurred 64 times, with a green dot on the surface of the chocolate bar, half of the time on the left side, and half on the right. Participants were instructed to press the trigger on the joystick as soon as they saw the dot appearing. For each participant, each chocolate bar was presented 16 times in a randomised order.

#### 2.5.3 Post-conditioning phase

Participants re-rated their explicit liking and wanting of each chocolate bar, and repeated the implicit motivation joystick approach-avoidance task where they were again shown the unlabelled chocolate bars.

#### 2.5.4 Final association test

After the above procedure was complete, HWL and IAL groups only were presented the unlabelled chocolate bars alongside the different labels they had been paired with, and participants were instructed to match which chocolate bar had been paired with which label.

#### 2.5.5 Post-study snack selection task

Finally, participants were offered a snack for immediate consumption. There were eight unlabelled snacks to choose from – the four energy-dense chocolate bars from the previous tasks and four healthier snacks. The eight snacks were presented together on a tray with the healthier and less healthy items mixed up in a consistent order. Participants were told to choose a snack to eat now or take away with them to eat at the end of the study.

Participants then completed a questionnaire, answering questions relating to their hunger level, time since last time they ate, demographics (education level, income level, age, gender), height and weight. Following this, participants were debriefed and reimbursed £30 for completing the study. The whole study session lasted approximately 30 min.

### 2.6 Analysis

A pre-specified statistical analysis plan was pre-registered on OSF (https://osf.io/pc4t6) prior to data analysis, with analysis conducted by a statistician who was not involved in the collection of the data.

GLMs (general linear model) were used to compare post-conditioning implicit motivation between the three study groups, with adjustment made for pre-conditioning implicit motivation. Two comparisons were made: (i) the difference in mean between HWL and IAL; and (ii) the difference in mean between HWL and IAL combined and the no label group. In unplanned analyses, participant characteristics of age, gender, education and BMI were additionally added to the primary outcome model as covariates. Conclusions were unchanged.

The change in implicit motivation (pre-conditioning implicit motivation minus post-conditioning implicit motivation) was also calculated. All errors (*i.e.* a participant pushed when they should have pulled, or vice versa) were recorded as −999 in the dataset and excluded before analysis. The GLM was repeated for both explicit liking and wanting post-conditioning scores, with adjustment for pre-conditioning scores. All mean differences are reported alongside a 95% CI, test statistic and p-value. Cohen's d is also reported for continuous outcomes. All model residuals were checked and were satisfactory in normality plots. GLMMs (generalized linear mixed model) were used as a sensitivity analysis of the raw data (repeated measurements within each participant), and model diagnostics were satisfactory.

Due to concerns of a floor effect, a chi-squared test was used to compare the proportion of ≥1 correct answers for associate learning (vs. 0 correct answers) between HWL and IAL groups. A chi-squared test was also used to compare the proportion of participants selecting a healthier snack (vs. a chocolate bar) between study groups. If a participant chose not to select any snack (n = 2) they were not included in this analysis. Since no effect of study group on snack selection was observed, mediation analysis was not conducted, as per the statistical analysis plan.

## 3 Results

In total, all 90 participants who were recruited completed the study (30 participants in each of the three study groups) and were included in the analysis. Participant characteristics are presented in Supplementary material S5. The mean age was 35.0 years old (SD [standard deviation] = 12.6) and 50% of the participants were male (n = 45). Due to concerns of slight imbalance between study groups, age, gender, education and BMI were added to the primary outcome model as covariates.

Raw means and estimated mean differences for all outcomes, between study groups, are shown in Table 1.

**Table 1:** Study 1: Post-conditioning outcome data between study groups.

Implicit motivation (ms)	Raw mean (SD)	Estimated^a^ MD(95% CI)	p value^a^	Cohens d^a^(95% CI)
IAL (n = 29^)	3.98 (16.03)	1.6 (−5.2, 8.3)	0.643	0.09 (−0.28, 0.46)
HWL (n = 29^)	4.88 (12.45)			
No label (n = 29^)	2.08 (8.93)	2.4 (−3.4, 8.1)	0.415	0.15 (−0.23, 0.52)
HWL/IAL combined (n = 58^)	4.42 (14.27)			
**Explicit liking**^b^				
IAL (n = 30)	59.15 (16.31)	2.3 (−4, 8.6)	0.467	0.13 (−0.23, 0.50)
HWL (n = 30)	63.88 (20.76)			
No label (n = 30)	58.49 (18.72)	1.95 (−3.5, 7.4)	0.476	0.13 (−0.23, 0.49)
HWL/IAL combined (n = 60)	61.51 (18.66)			
**Explicit wanting**^c^				
IAL (n = 30)	45.19 (21.36)	−2.1 (−8.7, 4.5)	0.525	−0.12 (−0.48, 0.25)
HWL (n = 30)	46.38 (21.54)			
No label (n = 30)	52.62 (24.01)	−4.3 (−10, 1.4)	0.136	−0.27 (−0.64, 0.09)
HWL/IAL combined (n = 60)	45.78 (21.28)			
**Associate learning**^d^	**0 correct, n (%)**	**≥ 1 correct, n (%)**	**Chi-sq value**	**p value**
IAL (n = 30)	16 (53.3)	14 (47)	4.05	0.044
HWL (n = 30)	8 (27.6)	21 (72)		
**Snack selection**	**Chocolate bar, n (%)**	**Healthy snack, n (%)**	**Chi-sq value**	**p value**
IAL (n = 30)	18 (66.7)	9 (33)	1.05	0.591
HWL (n = 30)	16 (53.3)	14 (47)		
No label (n = 30)	18 (60.0)	12 (40)		

^Three participants had no data for their implicit motivation, one from each study group, due to the fact that they made an error in 100% of their trials for an entire block (i.e. they had errors in 100% of their 48 approach and/or 48 avoid trials, in the pre-conditioning and/or post-conditioning phases). For all other outcomes n = 90.

Irrelevant Aversive Label (IAL). Health Warning Label (HWL). Mean difference (MD). Confidence interval (CI). Standard deviation (SD). Milliseconds (ms).

a With adjustment for pre-conditioning scores.

b Explicit liking responses measured on a 100 mm visual analogue scale, labelled at either end by ‘not at all’ (0), to ‘very’ (100).

c Explicit wanting responses measured on a 100 mm visual analogue scale, labelled at either end by ‘not at all’ (0), to ‘very’ (100).

d Matching chocolate bars to labels, possible scores ranged from 0 (no correct pairings) to 4 (all correct pairings).

### 3.1 Effects of labelling on implicit motivation (approach/avoidance joystick task)

There was no evidence of a difference in implicit motivation between the three study groups (p = 0.645, F(2, 83) = 0.440). Comparisons also showed no evidence of a significant difference in implicit motivation between the IAL and HWL groups (MD = 1.6 ms [−5.2, 8.3], p = 0.643) (Table 1), and no evidence of a difference in implicit motivation between the no label group and the IAL/HWL groups combined (MD = 2.4 ms [−3.4, 8.1], p = 0.415) (Table 1). The implicit motivation change scores can be seen descriptively in the Supplementary material (S6). In unplanned analyses of the raw avoid and approach times, conclusions were unchanged.

For both comparisons, three outliers (defined by any values that were at least 1.5 x inter quartile range [IQR] higher than the upper quartile) were removed for a sensitivity analysis. As an additional sensitivity analysis, the raw data was analysed with a mixed effects model for repeated-measures data. For both sensitivity analyses, the conclusions were unchanged.

244 trials (3% of all trials), from 28 participants, were recorded as an error in the implicit motivation task in either the pre-conditioning or post-conditioning phases. For further details see Supplementary material (S7).

### 3.2 Effects of labelling on explicit motivation (liking, wanting, snack selection)

There was no evidence of a significant difference in either explicit liking or wanting scores between the three study groups (p = 0.596, F [2,86] = 0.521 and p = 0.271, F [2, 86] = 1.326 respectively). Comparisons between study groups also showed no evidence of any significant differences and can be seen in Table 1.

Regarding snack selection, 39% of participants chose a healthier snack (vs. a chocolate bar). Although participants in the HWL group had the highest proportion of healthier snack selection, there was no evidence of a significant difference in snack selection between study arms (47% vs. 33% vs. 40%, χ^2^(2) = 1.05, p = 0.591) (Table 1).

### 3.3 Associative learning

Overall, the median number of correct pairings, out of a possible four, was one. There was some evidence for a significant difference in the number of correct pairings between study groups; participants in the HWL group were more likely to have ≥1 correct answers compared to the IAL group (72% vs. 47%, χ^2^(1) = 4.05, p = 0.044) (Table 1). We also intended to conduct an exploratory analysis including participants who had 3/4 or 4/4 correct pairings in the associate learning task. However, as only one participant had 4/4 correct pairings and none had 3/4 correct pairings, it was not possible to conduct this analysis.

## 4 Discussion

The results of Study 1 indicated no evidence of an effect of associating HWLs or IALs with chocolate bars on subsequent approach and avoidance towards the same chocolate bars when viewed in the absence of the labels, which was not consistent with our hypothesis that exposure to aversive labels (both HWLs and IALs) would reduce implicit motivation for energy-dense snacks. Nor did we find any evidence of the predicted increase in effect for HWLs compared to IALs.

In terms of more explicit measures, there was no evidence of a difference between the three conditions on liking and wanting scores, which was again inconsistent with the study hypothesis that exposure to aversive labels reduces explicit liking and wanting for energy-dense snacks, with greater reductions for HWLs than IALs. Finally, regarding snack selection, while participants in the HWL group had the highest percentage of healthier snack selection, there was no clear evidence of an effect of label condition on selection. Thus, there appear to be no measurable effects of aversive labelling, of either kind, on implicit and explicit measures of motivation.

There are several possible explanations for the results of Study 1. First, it is possible that the findings reflect insufficient power to detect other than large effects. In the absence of any salient prior studies to estimate a likely effect size, the study was powered on practical considerations, including laboratory recruitment and finances. Increasing the power of the study is therefore warranted before concluding an absence of effect.

Another important possibility is that the effects of exposure to the aversive labels on chocolate bars may only occur when the label is physically present at the time of response. The current study sought to establish an association (through the conditioning phase) in anticipation that subsequent presentation of the chocolate bar would re-evoke the associated label and exert an effect on motivation as a consequence. Thus, both implicit and explicit tasks depended on the establishment of an association. Given that post hoc assessment of associative knowledge showed poor explicit recall of chocolate-label pairings, despite the fact that pairings had been repeated 16 times for each pair, it remains unclear whether warning labels have to be present at the time of a measurement in order to exert their effects on that measure. With respect to choice behaviour, the evidence is mixed in regard to whether warning labels have to be present on packaging during that choice (*e.g.* Asbridge et al., 2021; Grummon & Brewer, 2020; Hollands & Marteau, 2016). Furthermore, these results may also indicate that 16 chocolate bar and label pairings were insufficient to fully establish an association between the chocolate bar and its corresponding label, and thus a higher number of chocolate bar-label pairings might be needed if a similar study design were to be used again.

These potential explanations informed the design of Study 2, which was conducted due to the absence of effects observed in Study 1. We used a much greater sample and ensured that motivation was tested in the presence of the labels (HWLs and IALs). The latter change is particularly relevant to real world effects since, if labels, such as HWLs, were to be used on food packaging in the real world, they would likely be present on the products during selection. For Study 2, we hypothesised that (i) exposure to aversive labels reduces implicit motivation for the labelled food, with reductions being greater for HWLs than IALs; and (ii) exposure to aversive labels reduces selection of energy-dense snacks, with this effect being greater for HWLs than IALs, and greater when products in the food selection task display aversive labels, compared to when they are unlabelled.

### 4.1 Study 2 – Online study of the effects of different aversive labels on concurrently measured motivation towards chocolate bars

As with Study 1, we compared the effects of HWLs and IALs. But we introduced two principal modifications: (i) using an experimental design with substantially increased power; and (ii) examining the effects of labelling on implicit motivation when the warning label was present on packaging, instead of relying on the evocation of its representation based on prior conditioning. Unlabelled neutral baseline stationery stimuli (pen, ruler, scissors, stapler) were used as comparison images in the approach-avoidance task, as described below. Note that stationery items were not associated with labels for any of the groups.

Due to COVID-19 restrictions, Study 2 was conducted online, requiring adaptation of the implicit motivation measure used in Study 1 to a manikin task. The manikin task is conceptually equivalent to the approach-avoidance joystick task used in Study 1, but more adaptable to a home setting as it only requires a keyboard. The snack selection measure from Study 1 was also used but in the form of hypothetical selection tasks.

The study protocol was preregistered on OSF (https://osf.io/xsfev); and on ISRCTN (https://doi.org/10.1186/ISRCTN16746619), prior to data collection. Ethical approval for this study was granted by the Cambridge Psychology Research Ethics Committee (reference: PRE.2019.111).

## 5 Methods

### 5.1 Design

In an online study, using a between-subjects design, participants were randomised to one of the three study groups used in Study 1: (i) HWLs; (ii) IALs; or (iii) no label. The study was run online using Qualtrics, Inquisit Web 4 and Inquisit Web 6 software.

### 5.2 Participants

A general population sample of 1382 adult participants – comprising similar proportions of men and women and similar proportions of lower and higher socio-economic position – was recruited via a research agency. Participants were over 18 years old, liked and consumed chocolate at least once a week, had basic computer literacy (*i.e.* able to use a computer for simple tasks) and internet access.

#### 5.2.1 Sample size determination

Given that running the study online meant that we were unable to use the same task as in Study 1, the required sample size could not be informed. Therefore, the current study was powered conservatively to detect a small effect (f = 0.10) between the three study groups in the primary outcome with power of 90% and an alpha level of 5%. This requires 423 participants per group, giving 1269 participants in total. In case of attrition between randomisation and outcome measurement, we increased the total by 5%, therefore aiming to randomise at least 1333 participants.

### 5.3 Materials

The same HWLs and IALs were used as in Study 1 (Fig. 1). To ensure the IALs matched the HWLs on ratings of negative emotional arousal, a general population survey was conducted (n = 256). The closest rated IAL to each HWL was selected. Full details can be seen in the Supplementary material (S8 and S9). The same four well-known chocolate bars were used as in Study 1. Photos of the baseline stationery stimuli used can be seen in Supplementary material S10.

### 5.4 Measures

#### 5.4.1 Primary outcome measure

##### 5.4.1.1 Implicit motivation: manikin task

The manikin task, comparable to the joystick task (which was unsuitable for an online study due to the requirement for a joystick), was used to quantify speed of response to either an “approach” or an “avoid” instruction in relation to images of chocolate bars and stationery stimuli using a manikin figure. Past research has found this measure to be reliable in demonstrating approach-avoidance effects (Krieglmeyer & Deutsch, 2010).

The manikin task comprised two blocks with a total of 96 trials: in one block of 48 trials participants had to approach the image if it depicted a chocolate bar and avoid the image if it depicted stationery stimuli. This rule was reversed in the other block of 48 trials. Whether the participant was asked to approach chocolate bars first, or avoid chocolate bars first, was randomised per participant and each block began with four practice trials. Chocolate bars appeared displaying labels (or no label) as per the participants’ study group. Depending on the instructions, participants had to press keys on their keypad (Y and B) to move a stick figure on the screen towards or away from images of chocolate bars and stationery (Supplementary material S11).

As in Study 1, implicit motivation for chocolate bars, was calculated for each participant by: mean avoid reaction time for chocolate bars minus mean approach reaction time for chocolate bars. A higher (or positive) implicit motivation value indicates more motivation to approach the chocolate bar. A lower (or negative) implicit motivation indicates less motivation to approach the chocolate bar.

Implicit motivation for stationery was also calculated from the manikin task, in the same way as it was for chocolate bars. Error rates from the manikin task were also collected.

#### 5.4.2 Secondary outcome measures

##### 5.4.2.1 Implicit motivation: go/no-go task

A subsequent go/no-go task was performed (after completion of the manikin task) and included two blocks, one in which chocolate bar images were assigned to a go trial and the stationery images to a no-go trial, and another where the rule was reversed. Chocolate bars appeared displaying labels (or no label) as per the participants’ study group. Each block contained 24 trials, of which 18 (75%) were go trials, and 6 (25%) were no-go trials. In one block participants were instructed to press the space bar as quickly as possible if they saw a chocolate bar (go trial), but inhibit this response if the image was of stationery (no-go trial), and vice versa (Supplementary material S12). The order of the two blocks was randomised, and each block began with four practice trials.

Three measures of implicit motivation were recorded from the go/no-go task: commission error rate (falsely making a go response by pressing the space bar in no-go trials), omission error rate (falsely not pressing the space bar in go trials) and mean response times for correct go responses, as seen in Loeber et al. (2012). A higher number of commission errors, a lower number of omission errors and a lower (therefore faster) reaction time indicate an approach bias, which in turn is indicative of a higher implicit motivation.

##### 5.4.2.2 Snack selection

Two sets of hypothetical snack selection tasks were completed, one after the manikin task and one after the go/no-go task. Selection tasks – including online food and drink selection – have been widely used in comparable studies (*e.g.* Clarke et al., 2020; Hollands & Marteau, 2016). The selection comprised eight items, including the four chocolate bars used in the previous tasks and four healthier snacks (apple, banana, orange, grapes) (Supplementary material S13). Participants were shown the selection and asked to select the food product they would most like to eat now. Both sets of selection tasks included one selection task in which the chocolate bars were unlabelled (in their original branded packaging) and a second selection task in which the chocolate bars were labelled depending on participants’ randomised study group (*i.e.* displaying HWLs, IALs, no labels). The healthier snacks always remained unlabelled.

##### 5.4.2.3 Explicit liking and wanting

A mean liking and wanting of the four chocolate bars and stationery stimuli was assessed using a 100 mm visual analogue scale, ranging from ‘not at all’ (0) to ‘very’ (100). The two questions used were based on standard measures used in studies of food and drugs (Rogers & Hardman, 2015):

Liking*: “*How much do you like this chocolate bar generally?” (chocolate bars)
“How much do you like this product generally?” (stationery stimuli)
Wanting: “How much do you want one of these chocolate bars right now?” (chocolate bars)
“How much do you want this product right now?” (stationery stimuli)

A mean liking and wanting score was calculated for the chocolate bars and the stationery stimuli. All explicit liking and wanting questions were asked at baseline and post-intervention (post manikin and go/no-go task).

#### 5.4.3 Additional measures

##### 5.4.3.1 Hunger

Assessed post-conditioning via a 100 mm visual analogue scale; “At the moment, how hungry are you?” (0; Not at all – 100; Very).

##### 5.4.3.2 Time since last ate

Assessed post-conditioning via a multiple choice question with 4 possible answers [Less than an hour ago/1–2 h ago/3–4 h ago/5 or more hours ago].

##### 5.4.3.3 Demographic characteristics

Assessed post-conditioning, including age, gender, ethnicity, highest education level, income level, height, and weight.

##### 5.4.3.4 Purchasing and consumption habits

Assessed via a two-item behaviour frequency measure used to assess strength of habit (Wood, Quinn, & Kashy, 2002): “How many times have you [purchased/eaten] this chocolate bar in the last month?” [Monthly or less often; At least once a week; Just about every day].

##### 5.4.3.5 Eating behaviour

Participants' eating behaviour; assessed via a three-item measure on cognitive restraint, from the Three Factor Eating Questionnaire (Cappelleri et al., 2009): “I deliberately take small helpings to control my weight.”, “I don't eat some foods because they make me fat.” and “I consciously hold back on how much I eat at meals to keep from gaining weight.” [Definitely true; Mostly true; Mostly false; Definitely false].

#### 5.4.4 Changes from protocol

We had also originally planned to address the following study aim: to assess how strongly different measures of explicit and implicit motivation predict selection behaviours. However, before the data were examined, we decided this was unlikely to be a helpful viable analysis given the study design as outlined in the pre-registered analysis plan (https://osf.io/xsfev).

### 5.5 Procedure

**Fig. 3:**
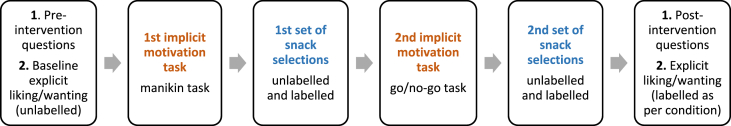
The flow of the steps of Study 2.

The flow of the procedure is illustrated in Fig. 3. Upon clicking on a link to start the study on the Qualtrics platform, participants were given the same cover story as in Study 1 and were informed that they were able to withdraw from the study at any time. They then provided online consent and were screened for eligibility via the Qualtrics software.

Participants started by answering questions on their hunger levels, time since they last ate and other demographic measures, and gave baseline measures of explicit wanting and liking for both the chocolate bars and stationery stimuli (all unlabelled). Next, participants were directed to a new webpage where they were instructed to download an Inquisit plug-in to complete the manikin task and were then randomised to their study group.

Immediately after the manikin task, participants were presented with two snack selection tasks of eight food items (the four chocolate bars and four healthier snacks) and asked to select the item they would most like to eat now. In the first selection task the chocolate bars were unlabelled (in their original branded packaging) and in the second the chocolate bars were labelled depending on participants’ randomised label condition (e.g. in the no label group the chocolate bars were not labelled).

Participants then completed the second implicit motivation task – the go/no-go task. They then repeated the snack selection tasks and the explicit liking and wanting ratings of the four chocolate bars and four stationery stimuli. Unlike the baseline explicit measures, this time the chocolate bars were labelled depending on participants’ randomised label condition. Lastly, participants answered questions on their eating behaviour, purchasing and consumption habits, height and weight.

### 5.6 Analysis

A comprehensive statistical analysis plan was agreed prior to inspection of the data (https://osf.io/xsfev), with analysis conducted by a statistician not involved in the collection of the data.

GLMs were used to compare the implicit motivation for chocolate bars between the three study groups. Adjustment was made for whether the participant was asked to approach or avoid chocolate bars first. All errors (where participants did the incorrect action to what they were asked to do, *e.g.* approached the chocolate bar when they should have avoided the chocolate and vice versa), as well as any trials with reaction times below 200 ms and above 1500 ms, were compared between groups and then excluded from the analysis. In unplanned analyses, participant characteristics of age, gender, education and BMI were also considered for model entry as covariates. Conclusions were unchanged.

Outcomes from the go/no-go task, were analysed in the same way as the primary outcome, and adjustment was made for whether the participant experienced blocks in which chocolate bar trials required go or no-go responses first. GLMs were also used to compare post-intervention mean explicit liking and wanting scores for chocolate bars between the three study groups. Adjustment was made for baseline mean explicit liking and wanting scores.

An umbrella (overall across all three study groups) p value and F statistic, and pairwise p-values between all three study groups, are reported. Pairwise comparisons between all three groups are also reported and a further comparison was made between the HWL and IAL groups combined against the no label group. All mean differences are reported alongside a 95% CI, test statistic and p-value. Cohen's d and f are also reported where appropriate.

All model residuals were checked and were satisfactory in normality plots for the primary outcome. Where model residuals were not satisfactory in normality plots, for outcomes from the go/no-go task (commission and omission errors), bootstrap P values from 1000 bootstrap samples, with 95% CI, were calculated. Results remained similar to the original analysis.

Selection task counts were analysed using a chi-squared test. To compare between percentages when labels are present vs. absent, z-statistics based on the 95% CI were used. Additional p values for comparing two proportions were calculated using the 95% CI and a 5%/6 (0.008) significant p value was adopted (Altman & Bland, 2011). Analyses comparing snack selection between HWL and IAL conditions were not pre-registered.

## 6 Results

1633 individuals were randomised into the three study groups (see Supplementary material S14 for more detail). 1382 successfully completed the study and were included in the analyses (HWL n = 457; IAL n = 454; no label n = 471). The mean age was 34.0 years (SD = 13) and 51% of the sample were female (n = 708). The majority of the participants reported having a higher education qualification (61%, n = 845). Participant demographic and baseline characteristics are presented in Supplementary material S15 and appear balanced between conditions.

Raw means and estimated mean differences for all outcomes, between study groups, are shown in Table 2. Raw means for all other measures, between study groups, can be seen in Supplementary material S16.

**Table 2:** Study 2: Primary and continuous secondary outcomes between study groups.

		Raw mean (SD)	Estimated MD compared to no label (95% CI)	Pairwise p value (compared to no label)	Cohens (95% CI)
**Implicit motivation score (ms) for chocolate bars – Manikin task**					
IAL (n = 447)	49.5 (123.5)		−30.5 (−47.5, −13.5)	<0.001	−0.23 (−0.36, −0.10)^d^
HWL(n = 453)	37.9 (145.8)		−42.0 (−59.0, −25.1)	<0.001	−0.32 (−0.45, −0.19)^d^
No label (n = 470)	80.9 (122.5)		-	-	0.14 (0.08, 0.19)^
HWL/IAL combined (n = 900)	43.7 (135.2)		−36.3 (−51.0, −21.7)	<0.001	−0.28 (−0.39, −0.16)^d^
**Rate of commission errors for chocolate bars - GNG task**					
IAL (n = 454)	0.19 (0.23)		−0.04 (−0.1, −0.01)	0.009	−0.17 (−0.30, −0.04)^d^
HWL(n = 455)	0.18 (0.20)		−0.05 (−0.1, −0.02)	0.001	−0.29 (−0.35, −0.09)^d^
No label (n = 470)	0.23 (0.22)		-	-	0.10 (0.04, 0.14)^
HWL/IAL combined (n = 909)	0.19 (0.21)		−0.04 (−0.1, −0.02)	<0.001	−0.20 (−0.31, −0.08)
**Rate of omission errors for chocolate bars - GNG task**					
IAL (n = 454)	0.03 (0.14)		0.01 (−0.01, 0.03)	0.244	0.08 (0.05, 0.21)^d^
HWL(n = 455)	0.02 (0.10)		−0.003 (−0.02, 0.01)	0.692	−0.03 (−0.15, 0.10)^d^
No label (n = 470)	0.02 (0.12)		-	-	0.04 (0.00, 0.09)^
HWL/IAL combined (n = 909)	0.03 (0.12)		0.003 (−0.01, 0.02)	0.656	0.03 (0.09, 0.14)^d^
**Reaction time (ms) for chocolate bars - GNG task**					
IAL (n = 454)	451.8 (77.6)		5.1 (−4.6, 14.8)	0.299	0.07 (0.06, 0.20)^d^
HWL (n = 455)	454.7 (73.7)		8.5 (−1.2, 18.2)	0.084	0.11 (0.02, 0.24)^d^
No label (n = 470)	446.5 (73.8)		-	-	0.05 (0.04, 0.16)^
HWL/IAL combined (n = 909)	453.2 (76.6)		6.8 (−1.5, 15.2)	0.109	0.09 (0.02, 0.20)^d^
**Explicit liking**^a^					
IAL (n = 454)	56.7 (23.4)		−8.0 (−10.0, −6.0)	<0.001	−0.51 (−0.64, −038)^d^
HWL (n = 457)	57.3 (23.3)		−8.9 (−10.9, −6.9)	<0.001	−0.57 (−0.70, −0.44)^d^
No label (n = 471)	64.2 (19.0)		-	-	0.26 (0.20, 0.31)^
HWL/IAL combined (n = 911)	57.0 (23.3)		−8.4 (−10.2, −6.7)	<0.001	−0.54 (−0.65, −0.43)^d^
**Explicit wanting**^b^					
IAL (n = 454)	34.5 (27.3)		−8.4 (−10.7, −6.2)	<0.001	−0.48 (−0.61, −0.35)^d^
HWL (n = 457)	30.7 (27.0)		−12.2 (−14.5, −9.9)	<0.001	−0.69 (−0.82, −0.56)^d^
No label (n = 471)	42.2 (27.5)		-	-	0.29 (0.23, 0.34)^
HWL/IAL combined (n = 911)	32.6 (27.2)		−10.3 (−12.3, −8.4)	<0.001	−0.58 (−0.69, −0.47)^d^
**Implicit motivation score (ms) for stationery – manikin task**^c^					
IAL (n = 447)	19.8 (120.9)		21.2 (4.9, 37.5)	0.011	0.17 (0.04, 0.30)^d^
HWL (n = 453)	13.0 (140.8)		14.4 (−1.8, 30.7)	0.082	0.11 (0.01, 0.24)^d^
No label (n = 470)	−0.7 (113.8)		-	-	0.07 (0.00, 0.12)^
HWL/IAL combined (n = 900)	16.4 (124.7)		17.8 (3.8, 31.8)	0.013	0.41 (0.29, 0.52)

Milliseconds (ms). Irrelevant Aversive labels (IAL). Health Warning Label (HWL). Confidence interval (CI). Standard deviation (SD). difference (MD). Confidence interval (CI). Go/no-go (GNG). Milliseconds (ms).

N.B. 12 participants had missing data for the manikin task and 3 participants had missing data for the go/no-go task, all other analysis is complete.

^ Cohen's f between all three groups.

a Explicit liking responses measured on a 100 mm visual analogue scale, labelled at either end by ‘not at all’ (0), to ‘very’ (100).

b Explicit wanting responses measured on a 100 mm visual analogue scale, labelled at either end by ‘not at all’ (0), to ‘very’ (100).

c Stationery items did not have labels on them and were presented in the HWL, IAL and no label groups as control stimuli.

d Cohen's d compared to the no label group.

### 6.1 Effects of labelling on implicit motivation (manikin task, go/no-go)

There was evidence of a significant difference in implicit motivation for chocolate bars between the three study groups in the manikin task (p < 0.001, F [2, 1366] = 12.7). There was evidence of a significant reduction in implicit motivation for chocolate bars in the HWL group (MD = −42.0 ms [−59.0, −25.1], p < 0.001), and the IAL group (MD = −30.5 ms [−47.5, −13.5], p < 0.001) compared to the no label group (Table 2). When the IAL and HWL groups were combined there was also a significant reduction in implicit motivation for chocolate bars (MD = −36.3 ms [−51.0, −21.7], p < 0.001), compared to the no label group (Table 2). In unplanned analyses of the raw avoid and approach times, conclusions were unchanged. The difference between groups was primarily driven by approach reactions taking longer when labels were present.

During the go/no-go task, there was also evidence of a significant reduction in implicit motivation for chocolate bars, indicated by lower commission error (falsely making a go response by pressing the space bar in no-go trials) rates, in the IAL (MD = −0.04 [−0.07, −0.01], p = 0.009) and HWL groups (MD = −0.05 [−0.08, −0.02], p = 0.001) compared to the no label group (Table 2). There was evidence of a significant reduction in implicit motivation for chocolate bars, again indicated by lower commission error rates, when the HWL and IAL groups were combined (MD = −0.04 [−0.07, −0.02], p < 0.001) compared to the no label group (Table 2). Omission error (making a no-go response by not pressing the space bar in go trials) rates and reaction times in the go/no-go task showed no evidence of a significant difference between groups (Table 2).

In terms of differences between the two warning aversive label types, there was no clear evidence of a significant difference in implicit motivation for chocolate bars between the HWL and IAL groups in the manikin task (MD = 11.5 [−5.6, 28.6], p = 0.188). There was also no evidence of a significant difference in commission error rates, omission error rates or reaction times between the IAL and HWL groups in the go/no-go task (p = 0.492, p = 0.122 and p = 0.497 respectively). Thus, for the set of measures signifying implicit motivation towards snacks, the impact of HWLs and IALs (alone and combined) was significant compared to no label but did not differ across the two label types.

For the stationery stimuli, there was evidence of a significant difference between the no label group and the IAL group in implicit motivation score in the manikin task (MD = 21.2 [4.9, 37.5], p = 0.011) (Table 2). This evidence was not seen for the difference between the HWL and the no label group (MD = 14.4 [−1.8, 30.7], p = 0.082) (Table 2). There was no evidence of a difference in the implicit motivation for stationery stimuli between the HWL and IAL groups (p = 0.419).

In the manikin task, there was no evidence of a main effect of whether the participant was asked to approach or avoid chocolate bars first. In the go/no-go task, there was evidence of a main effect for whether the participant experienced blocks in which chocolate bar trials required go or no-go responses first for reaction times only (p = 0.005). Examining the interaction term between this order of go/no-go and study group showed that the order of go/no-go tasks was only affected in the IAL group.

Outliers (n = 83) (defined as any value that differs from the median by more than 3 units using MAD [median absolute value]) and seven participants who were recorded as having >50% errors in the manikin task were removed for a sensitivity analysis of the above analysis. Results remained similar.

Errors in the manikin task can be seen in Supplementary material S16. There were 9277 errors (7% of total errors) recorded from 1276 participants.

### 6.2 Effects of labelling on explicit motivation (snack selection, liking, wanting)

**Fig. 4:**
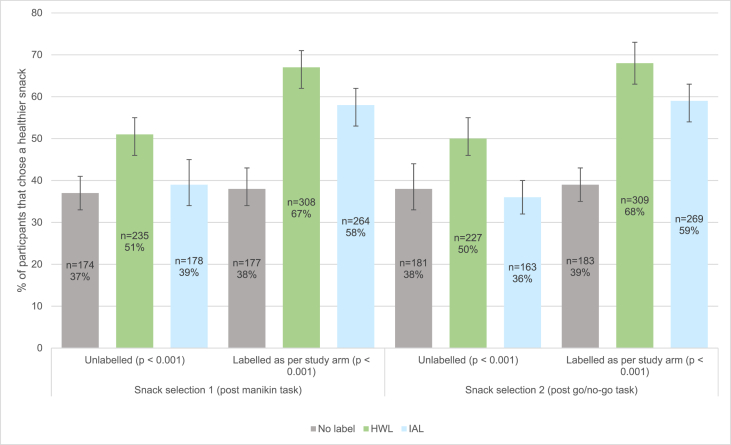
Study 2: Healthier snack selection between study groups. Error bars are 95% CI. P values are between study groups. Health Warning Label (HWL). Irrelevant Aversive label (IAL).

There was evidence of a significant difference in the proportion of healthier snacks selected between the three study groups for all four snack selections (all p < 0.001), with the HWL group consistently selecting the highest number of healthier snacks (Fig. 4) (Supplementary material S17). Carrying out the analyses according to whether a label was present or absent at the time of selection showed the following effects:

#### 6.2.1 Selection with label present

There was evidence of a significantly higher healthier snack selection in the HWL group compared to the IAL group in snack selection 1 for labelled snacks (67% vs. 58%, χ^2^(1) = 8.33, p = 0.004) (Fig. 4). There was no clear evidence of a significant difference in the proportion of healthier snacks selected between the HWL and IAL groups for snack selection 2 for labelled snacks (5%/6 = 0.008 Bonferroni adjusted threshold for significance) (68% vs. 59%, χ^2^(1) = 6.87, p = 0.009) (Fig. 4) (Supplementary material S18a).

#### 6.2.2 Selection with label absent

In selection task 1, when the chocolate bar was unlabelled, there was evidence of a significantly higher healthier snack selection in the HWL group compared to the IAL group (51% vs. 39%, χ^2^(1) = 13.71, p < 0.001) (Fig. 4). In selection task 2, again when the chocolate bar was unlabelled, this was similar: 50% in the HWL group and 36% in the IAL group (χ^2^(1) = 17.64, p < 0.001) (Fig. 4).

#### 6.2.3 Selection with label present vs. absent

In selection task 1, there was evidence of a significantly higher proportion of healthier snacks selected by participants when the chocolate bars were labelled compared to when they were unlabelled for both the HWL group (67% vs. 51%, p < 0.001) and IAL group (58% vs. 39%, p < 0.001) (Fig. 4). This pattern was the same for selection task 2: HWL group (68% and 50% respectively, p < 0.001) and IAL group (59% and 36% respectively, p < 0.001) (Fig. 4) (Supplementary material S18b).

In brief, both HWLs and IALs had greater effects when labels were present at the time of selection. However, HWLs produced significantly stronger effects both when labels were present and when they were not.

#### 6.2.4 Liking and wanting

There was evidence that wanting of chocolate bars was significantly lower in the HWL (MD = −12.2 [−14.5, −9.9], p < 0.001) and IAL (MD = −8.4 [−10.7, −6.2], p < 0.001) groups, compared to the no label group (Table 2). This was repeated for liking of the chocolate bar in the: HWL (MD = −8.9 [−10.9, −6.9], p < 0.001) and IAL group (MD = −8.0 [−10.0, −6.0], p < 0.001). There was no evidence of a significant difference in the mean liking of chocolate bars between the IAL and HWL groups (p = 0.394). However, there was evidence of a significantly lower mean wanting of chocolate bars in the HWL group compared to the IAL group (MD = −4 [−2, −6], p < 0.001). Therefore, for the set of measures signifying explicit motivation towards snacks, the impact of HWLs and IALs (alone and combined) was significant compared to no label and it also differed across the two label types, with HWLs having a greater impact than IALs.

## 7 Discussion

The findings from Study 2 indicate that both types of aversive label – HWLs and IALs – presented on packaging reduce implicit motivation towards energy-dense snack foods. This effect was observed in both the manikin and the go/no-go tasks, which further confirms the primary hypothesis that exposure to aversive labels reduces implicit motivation for the labelled food. However, this reduction in implicit motivation was not found to be greater when chocolate bars displayed HWLs compared to IALs in either implicit motivation task, which is inconsistent with our second hypothesis that reduction in implicit motivation is greater for HWLs than IALs.

Similar to the effects of labelling on implicit motivation, the results from explicit measures also indicated that exposure to both aversive labels reduces motivation, as indicated by liking, wanting and selection of energy-dense snack foods. Notably, unlike the implicit motivation tasks, the health-relevance of the label was a significant factor for two of the measures: snack selection and subjective wanting. In the first snack selection task healthier snack (i.e. fruit) selection was higher when participants were presented with chocolate bars that had HWLs on their packaging compared to IALs. Although the results went in the same direction in the second selection task, the difference between HWLs and IALs was not statistically significant. Furthermore, within-group comparisons in both selection tasks showed that when chocolate bars were labelled with either HWLs or IALs, a higher number of healthier snacks were selected compared to during the unlabelled selection tasks in the same groups. These findings from selection behaviour measures are in line with our hypotheses that labels, particularly HWLs, lead to healthier snack choices. Interestingly, it was found that even during unlabelled snack selections (right after implicit motivation tasks), a higher number of healthier snacks was selected by participants in the HWL group compared to IAL group. This suggests that in the absence of aversive labels, HWLs have a greater lingering effect than IALs following exposure to labels during the implicit motivation tasks.

Explicit liking and wanting scores for chocolate bars were also lower in both label groups compared to the no label group. While there was no statistical difference in mean liking scores between the HWL and IAL groups, participants in the HWL group reported wanting the chocolate bars less than those in the IAL group. Altogether these findings from explicit measures (*i.e.* snack selection, liking, wanting) indicate that both aversive labels are more effective at reducing the selection of energy-dense snack foods than no label. Apart from the liking measure, HWLs were shown to be more effective than IALs at reducing explicit motivation for unhealthy snacks.

## 8 General discussion

Deepening the understanding of the underlying mechanisms of warning labels would help to optimise their effectiveness in real-world settings. Two studies were conducted to assess whether labelling effects on motivation arise from the creation of an outcome-dependent association between the food and its health-harming consequences or from a simple aversive association. Findings across the two studies show first that both types of labels reduce implicit and explicit motivation towards energy-dense snack foods but, with some exceptions relating to explicit measures, the labels need to be present to produce a measurable effect. Second, there were no differences between HWLs and IALs in impact on implicit motivation. This suggests that the mechanism of action on implicit behaviour may be a simple association-based devaluation of the snack, rather than the production of a new outcome-dependent association between the snack and a health-related outcome. However, as HWLs were more effective at reducing energy-dense snack selection and subjective wanting than IALs, aversive warning labels may influence explicit choice-related behaviours by depicting a causal relationship between the product and the outcome.

The findings from our implicit measures support previous research that has investigated the effect of HWLs on implicit attitudes and dietary self-control (Asbridge et al., 2021; Hollands & Marteau, 2016; Rosenblatt, Bode, et al., 2018; Rosenblatt, Summerell, et al., 2018), and demonstrate that HWLs can additionally decrease implicit motivation for unhealthy snack foods. Measures of motivation have been suggested to be more useful in determining behaviour than measures of implicit attitudes as, in reflecting approach tendencies, they are more closely linked to the actual behaviour (Meissner, Grigutsch, Koranyi, Müller, & Rothermund, 2019). While the results from choice-related explicit measures align with Clarke et al. (2020; 2021c), Hollands and Marteau (2016) and Grummon et al. (2019) – who also showed that HWLs lead to healthier dietary choices – they are not in line with Asbridge et al. (2021), where the type of HWL did not affect food choice or explicit attitudes. One possibility could be that as the aversive labels were not present on packaging during the snack selection task, just like in Study 1 of the current experiment, prior exposure to the labels did not have a lasting effect that could have led to healthier food choices. This is in contrast to the study by Hollands and Marteau (2016) which did not display labels on packaging at the time of selection and still found a significant effect on snack choice. The conclusion from our studies and from existing work (Clarke et al., 2021a; Grummon & Brewer, 2020; Grummon et al., 2019) – some of which has been conducted in more naturalistic settings – is that, there may be an effect, albeit an attenuated one, when labels have been pre-exposed but are not present at the time of testing, but stronger effects may be observed when the labels are present. It also seems likely that differences in findings across studies may be influenced by various factors (*i.e.* different settings, measures, and study designs). Certainly, our own findings attest to the importance of task demands in revealing the influence of different mechanisms underlying label effects. Importantly, foreseeable real-world implementation is most likely to entail the concurrent presence of warning labels (as is the case with cigarette packs) and our findings support this as the optimal approach.

A potential advantage of examining the mechanisms of behaviour change lies in its potential for exploiting evidence from basic neuroscience in developing and optimising interventions aimed at producing such changes (Marteau, Fletcher, Hollands, & Munafò, 2020). While this is necessarily speculative, it is worth considering the current findings in the context of the neuroscience of learning and decision-making. Here, a distinction can be made between model-free and model-based behaviours (Dayan & Berridge, 2014; Sutton & Barto, 2018). This distinction itself relates to, though it does not completely overlap with, a longer-standing one between stimulus-driven (habitual) and goal-directed responding (De Wit & Dickinson, 2009). According to a model-free account, a decision or behaviour can be driven as a simple response to a stimulus, one that does not entail an explicit representation of the outcome or the wider impact that this behaviour may have. Such behaviours tend to be relatively automatic and unreflective. Conversely, model-based behaviours explicitly represent the decision/behaviour within a wider context and entail representation of outcomes. They are therefore more reflective and flexible. It is also important to acknowledge that the distinction between model-free and model-based learning and decision making – as with the strongly overlapping distinction between habit-based and goal-directed behaviours – does not reflect a straightforward dichotomy and it appears that individuals call upon both to varying degrees depending upon context and demands (Lee, Shimojo, & O'Doherty, 2014; O’Doherty, Cockburn, & Pauli, 2017).

It is plausible that a causal link between consumption and outcome, as is depicted by the HWLs, could exploit both model-free and model-based processing while a non-causally related label (*i.e.* IAL) would only engage model-free changes. This would be in keeping with the HWLs producing more wide-ranging effects, on both implicit and explicit motivational behaviours. It should be acknowledged that this is speculative and further experimental work, along with tailored computational modelling approaches would be required to develop the idea. Nonetheless, there are at least two reasons why distinguishing these mechanisms may prove useful and important. The first, as outlined, concerns the evidence that the behaviours differ in terms of their flexibility and persistence. The second concerns the evidence that the behaviours operate differently in varying contexts. For example, habitual or model-free behaviours require minimal attentional engagement and may be highly persistent even when the outcome becomes undesirable. They may be promoted by increased stress or reduced time. In addition, there is some evidence that model-free and model-based behaviours relate to dissociable, though overlapping, neural systems (Daw, Gershman, Seymour, Dayan, & Dolan, 2011; Lee et al., 2014). While the latter may not be of primary interest to the behavioural scientist or policymaker, if the science of behaviour change is to benefit from neuroscience, and vice-versa, linking interventions to underlying neurobiological processes may prove valuable (Marteau et al., 2020).

Leaving this speculation aside, we conclude that, although the aversive component of labels has a strong effect, emphasizing the causal relationship between the product and its health effects may be the key to targeting unhealthy food consumption. However, it cannot be assumed that everyone exposed to HWLs will already have a clear causal model linking the health outcome with the target behaviour. For example, a lack of awareness has been observed for the link between alcohol consumption and different types of cancers (Buykx et al., 2016). As such, ensuring that the causal relationship between the product and the adverse health outcome is clear may be important in eliciting behaviour change. In the current study, we used additional text on the label – as is the case for current tobacco HWLs – to emphasise the link. There may be ways to ensure that the image and the text are combined optimally on a label to relate both an aversive and an informative effect, maximizing the impact.

### 8.1 Strengths and limitations

This study's protocol and statistical analysis plan were pre-registered ahead of the launch of the trial and analysis respectively. The analyses were conducted by blinded statisticians who were unaware of condition allocation. Another strength of the study was the sample size of Study 2. Due to COVID-19 restrictions, Study 2 was conducted online which allowed a much larger sample to be recruited than for Study 1. Both studies had a relatively balanced sample regarding gender and education levels, recruited purposefully from the general population, with an exception that Study 2 had a slightly higher proportion of participants who were highly educated. Another key strength of this research was piloting undertaken in Study 1 and 2 to ensure that HWLs and IALs were equally aversive to avoid bias of one type of label (*e.g.* IALs being more aversive due to the nature of their images) over the other.

One of the main limitations was the sample size of Study 1, which may have not had enough power to detect small effects. This is a potential explanation for why no significant effects of labels were observed in contrast to Study 2, which had a much larger sample size to detect effects. However, Study 1 and 2 used different tasks (approach-avoidance task vs. manikin and go/no-go tasks) and methods (joystick vs. keyboard) to measure implicit motivation, and thus the results from the two studies can only be compared with reservation.

While there are positives about conducting Study 2 online, the main limitation regarding online studies is the lack of control over the setting the participant is in or the level of attention the participant pays during the experiment. However, as this was a randomised study comprising a large sample, such effects are expected to balance out across the study. Another limitation is a high drop-out rate post-randomisation. In Study 2, the high drop-out rate post-randomisation was most likely due to issues with plug-in downloads for both implicit motivation tasks. This feedback was given directly by some of the participants and the research agency who recruited the participants.

It is also important to highlight the differences in the nature of the images for HWLs (adverse health conditions influenced by excess calorie consumption) and IALs (dead/injured/aggressive animals). The labels inevitably differ in more than whether they depict a causal or non-causal link to overconsumption, a cause for caution. However, it would be highly challenging to achieve a perfect comparison whereby the HWLs and IALs depict similar things while also being clearly health-relevant or irrelevant. An example would be IALs also depicting adverse health problems, but those that are not influenced by excess calorie consumption, such as a cut or a broken arm. In this case one could not be sure that a non-conscious causal link to health would not be made by participants exposed to IALs. Nevertheless, disentangling causal and non-causal labels would be of interest for future research.

Another limitation to consider is that the second set of snack selections after the second implicit motivation (go/no-go) task in Study 2 – there to examine the effect of exposure to labels in the go/no-go task – may have been influenced by the preceding implicit motivation (manikin) task. If the latter produced lingering effects, then the second set of snack selection measures may have been influenced not just by the go/no-go task but by the manikin task as well. While acknowledging this, we suggest that the first set of snack selection tasks can be interpreted more specifically in terms of the influence of the labels presented during the manikin task.

### 8.2 Future research directions

Identifying underlying mechanisms important in shaping how warning labels act is ultimately only useful insofar as it allows us to predict and optimise those influences. We therefore see these findings as a prelude to closer interactions between behavioural sciences and relevant areas of cognitive neuroscience. It would be useful, for example, to examine, the effects of specific manipulations to labels with a view to predicting their effects on different types of behaviour in the laboratory setting. Indeed, neurophysiological measures of the effects of HWLs are already under study (Rosenblatt, Summerell et al., 2018). Moreover, growing understanding of how relevant neural systems and associated behaviours dissociate under stress, time pressure, hunger, and other factors that are highly relevant to real world behaviours and experiences, can feed potentially important information into planned interventions.

While we have emphasised the creation of associations between warning labels and snack foods, we should also be mindful that some participants may have pre-existing associations between energy dense foods and ill-health. It may be that HWLs therefore do not necessarily create such associations anew but rather activate their representations. Clarifying this would require further research.

Although the findings of this study demonstrate that aversive labels reduce implicit and explicit motivation for snacks, the relative importance of the image and text components of the labels was not possible to determine due to the study design. Previous research has found that adding text to the labels could be the principal component affecting implicit attitudes, while image-only labels did not have an impact (Asbridge et al., 2021). The effect of labels on motivation seen in the current study could also potentially be primarily driven by the text component of the labels. Disentangling the effects of image and text of HWLs and IALs in future research is therefore necessary for the optimisation of labels to increase their effectiveness. While research into IALs is unlikely to have any direct practical application in real world settings, it could have important broader theoretical implications.

## 9 Conclusion

The current study found evidence that both types of aversive label – HWLs and IALs – reduced implicit motivation toward energy-dense snack foods, with no significant difference between them. Both types of label also reduced explicit motivation for snacks, with this effect particularly pronounced with HWLs. Therefore, aversive HWLs appear to act both through low level associative mechanisms affecting implicit motivation, and by additionally emphasizing explicit causal links to health outcomes thereby affecting explicitly motivated choice behaviours. These findings provide an insight into mechanisms that underlie the effect of warning labels, which can inform future public health interventions.

## Funding

This research was funded in whole, or in part, by the 10.13039/100010269Wellcome Trust [ref:206853/Z/17/Z]. **For the purpose of Open Access, the author has applied a CC BY public copyright licence to any Author Accepted Manuscript version arising from this submission.**

## Ethics approval

Approved by the Psychology Research Ethics Committee of the University of Cambridge (reference: PRE.2019.015 [Study 1] and PRE.2019.111 [Study 2]). All research was performed in accordance with the Declaration of Helsinki.

## Consent to participate

Written informed consent was obtained from participants for Study 1 and an informed online consent was obtained from participants for Study 2.

## Consent for publication

Not applicable.

## Availability of data and materials

The datasets generated and analysed during the current study are available on the Open Science Framework project page (https://osf.io/xsfev) and the Cambridge University Repository (https://doi.org/10.17863/CAM.84729).

## Code availability

Analysis was conducted in SPSS v26 and v27. Analysis code, datasets and metadata are available on the Open Science Framework project page (https://osf.io/xsfev) and the Cambridge University Repository (https://doi.org/10.17863/CAM.84729).

## Authors' contributions

P.C.F., G.J.H., T.M.M., E.P. and M.V. contributed to the conception and design of the study protocols. E.P. and M.V. managed day-to-day running of the study. G.M., H.Z., E.P. and M.V. helped with setting up the experiments. K.D-L., M.A.P. and R.W.M. conducted data analysis, and helped with data interpretation together with P.C.F., G.J.H., M.V., E.P. and T.M.M. This manuscript was written by E.P., M.V. and P.C.F. with input from all co-authors. All authors read and approved the final version of the manuscript.

## Declaration of competing interest

The authors declare that they have no competing interests.

## Abbreviations

CI: Confidence interval
GLM: General linear model
GLMM: Generalized linear mixed model
GNG: Go/no-go
HWL: Health warning label
IAL: Irrelevant warning label
ISRCTN: International Standard Randomised Controlled Trial Number
IQR: Inter quartile range
MAD: Mean absolute value
MD: Mean difference
MS: Millisecond
OSF: Open Science Framework
SD: Standard deviation
