# Supplementary material for: Effect of health warning labels on motivation towards energy-dense snack foods: Two experimental studies
Source: Appetite. 2022 Aug 1;175:106084. doi: 10.1016/j.appet.2022.106084 (PMC9194909; doi:10.1016/j.appet.2022.106084)
Supplement: Multimedia component 1 [file mmc1.docx]

**S1. Study 1. Development of stimuli – Health warning labels**

Four HWLs (health warning labels) were selected as those which elicited the highest levels of negative emotional arousal in a large online study assessing responses to images depicting aversive consequences of consumption (Pechey et al., 2020)^[[1]](#footnote-1)^. Images depicted adverse health conditions (e.g. eye problems due to type 2 diabetes, surgery relating to bowel cancer, or heart disease) and were presented together with a warning text statement about the negative health consequences of excess calorie consumption. See below for examples.


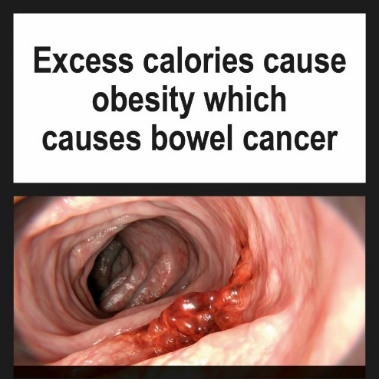

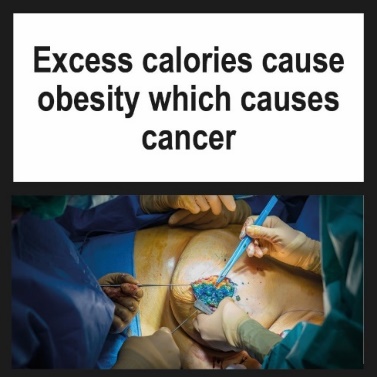


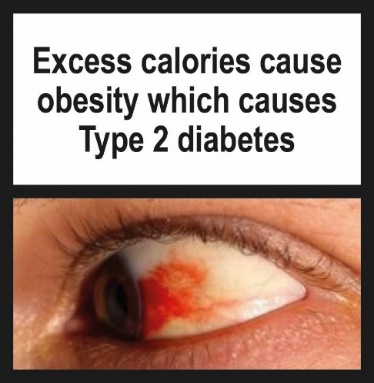

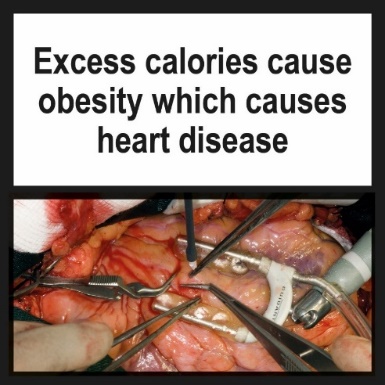


HWLs on chocolate bars:


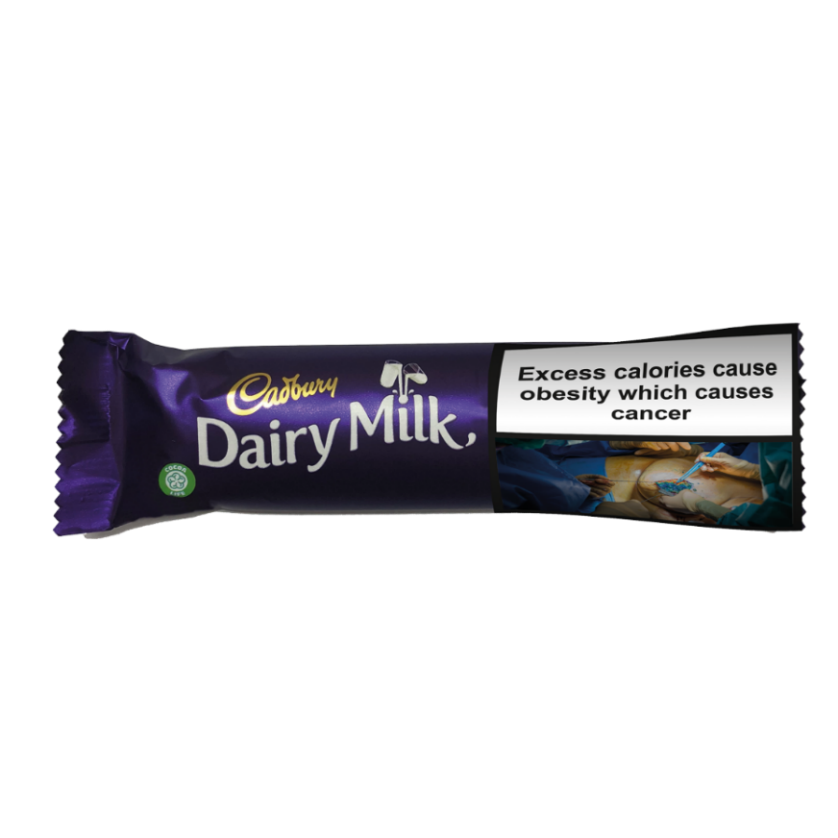

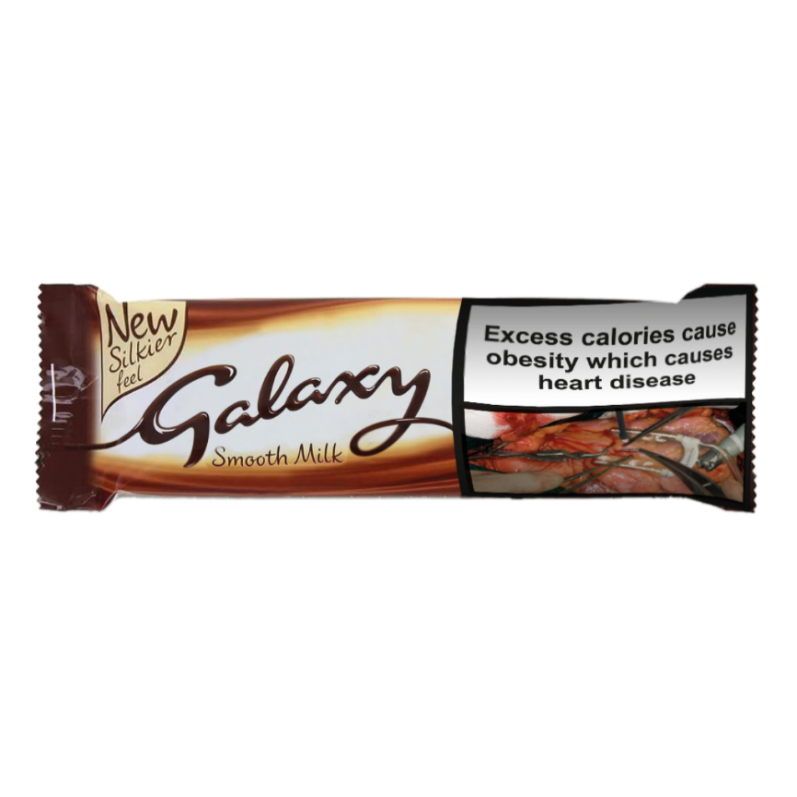

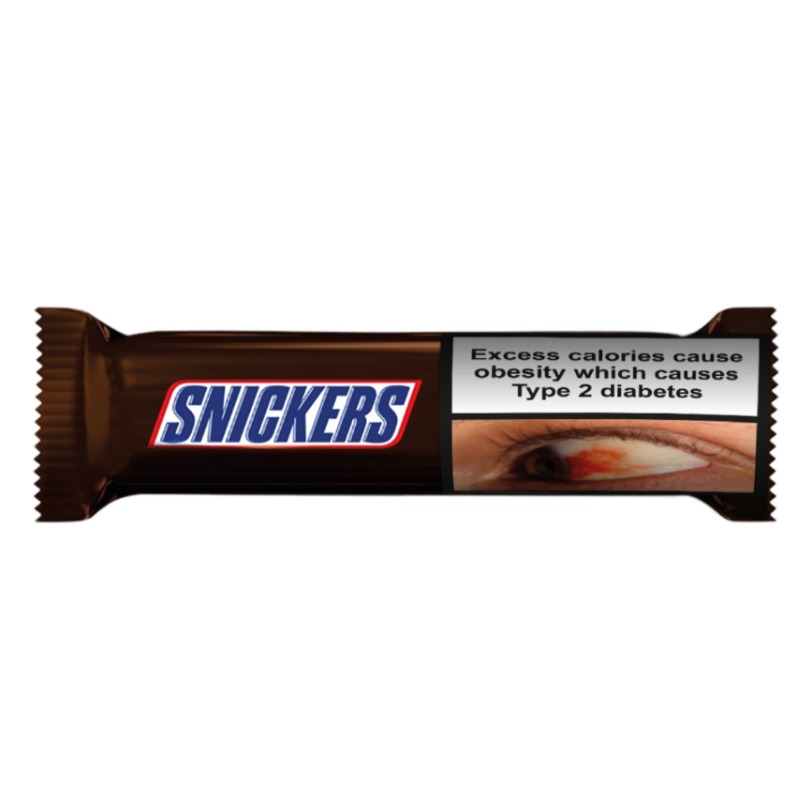

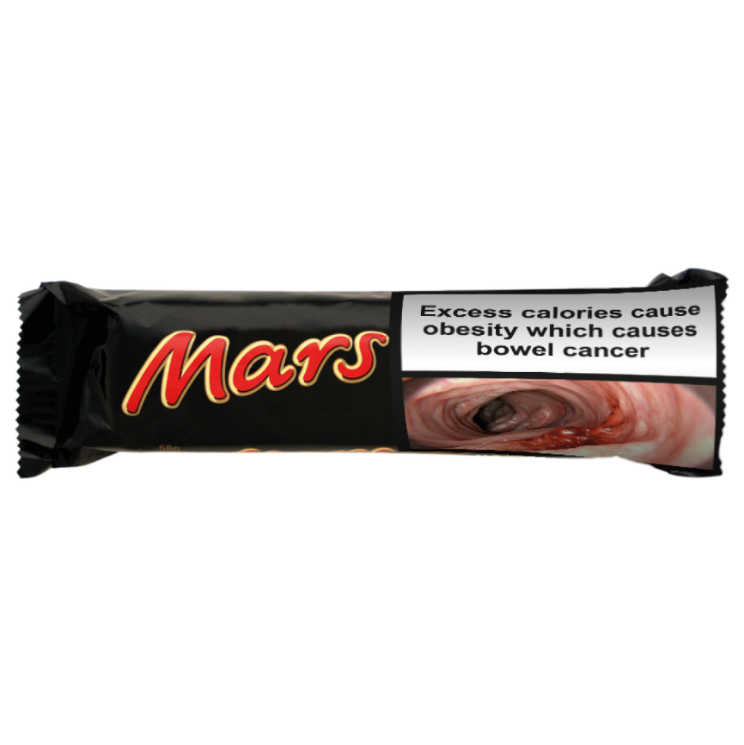


### S2. Study 1. Development of stimuli – Irrelevant aversive labels

Four IALs (irrelevant aversive labels) were created using four images selected from the International Affective Picture System (see https://csea.phhp.ufl.edu/media/ iapsmessage.html) which is a widely used database containing validated and standardised positive, negative and neutral images. We selected negative images that form a coherent grouping in terms of content, such as dead, injured or aggressive animals that are distinct in being irrelevant to chocolate bar consumption and to the relevant category stimuli (*i.e.* they do not portray human figures, or injuries or disease in people). The four images were selected via an internal pilot with colleagues (n=11), whereby a selection of irrelevant animal-based images were circulated and rated for negative emotional arousal. The irrelevant images with the most similar ratings of negative emotional arousal to the relevant HWLs were selected. In order to match the content of the HWLs, the IALs included a text statement that explains the content of the image using a comparable amount of words as for the HWLs. See below for examples.


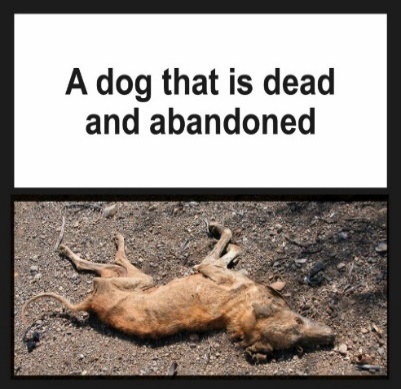

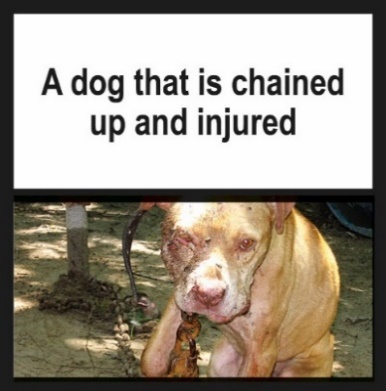

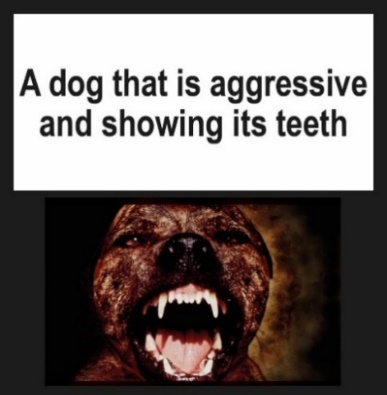

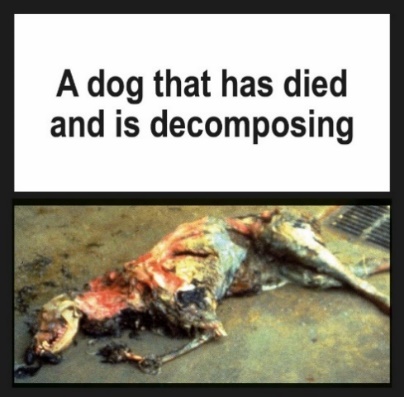


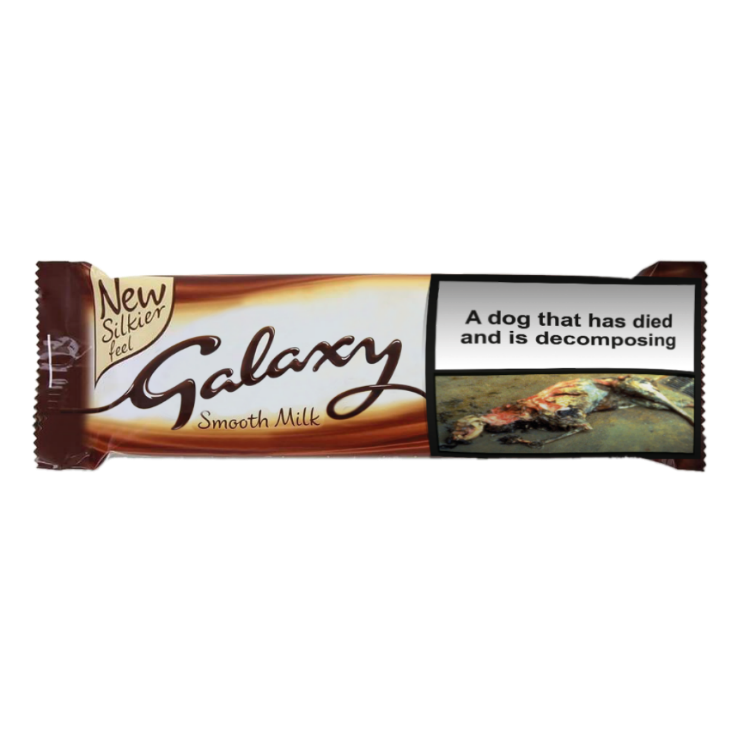
IALs on chocolate bars:


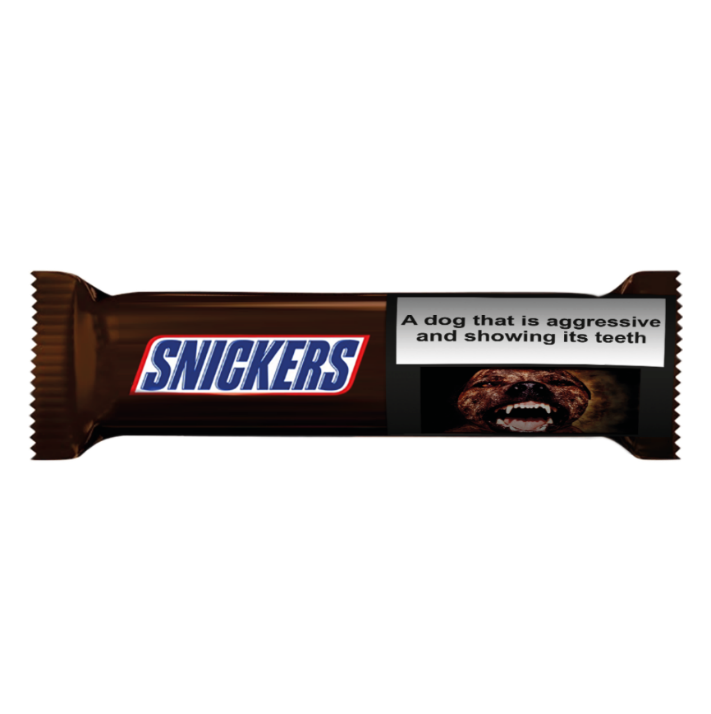


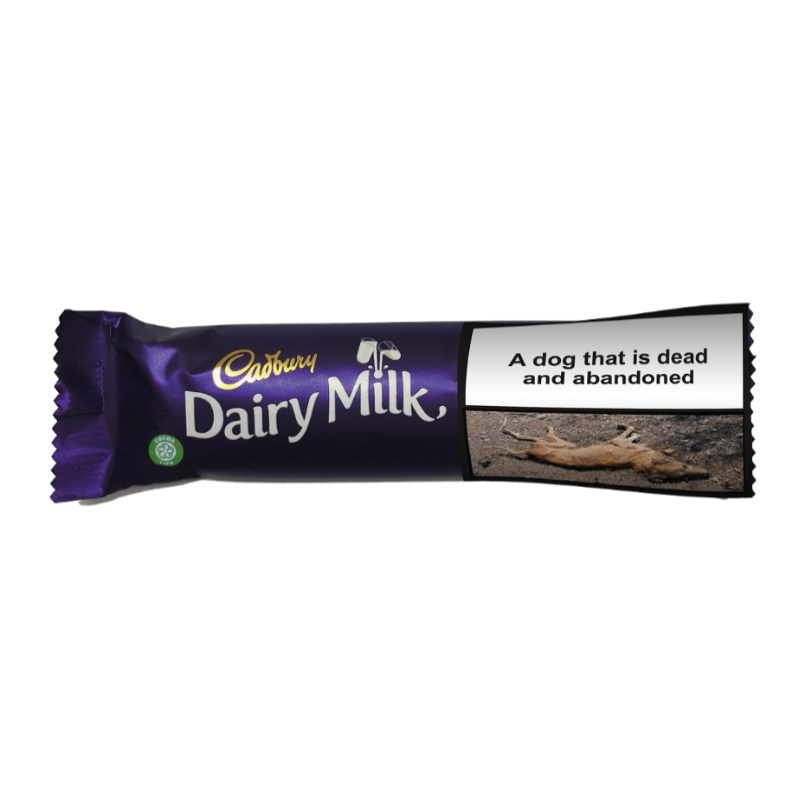


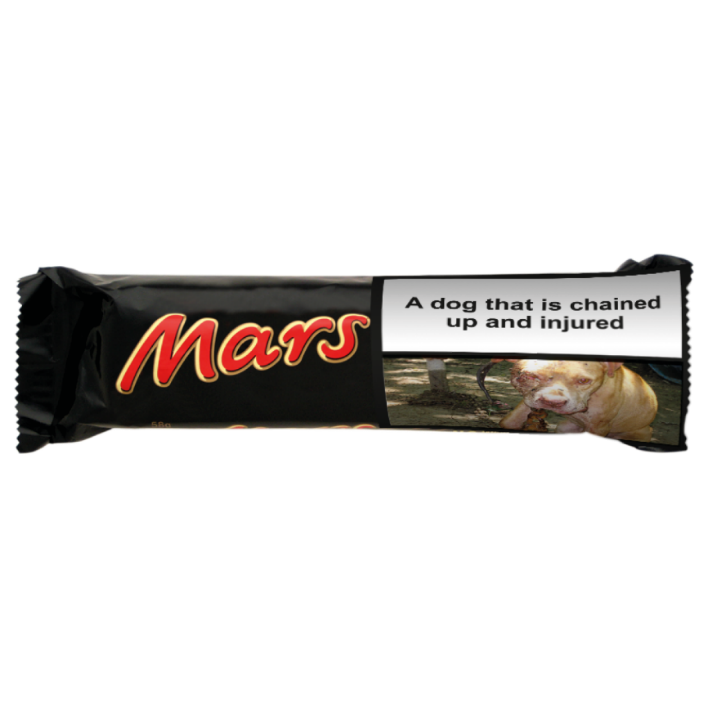


**S3. Study 1.** **Implicit motivation: joystick task**

Figure S11 (A): The “avoid” response which resulted in shrinkage of the image

Figure S11 (B): The “approach” response resulted in enlargement of the image


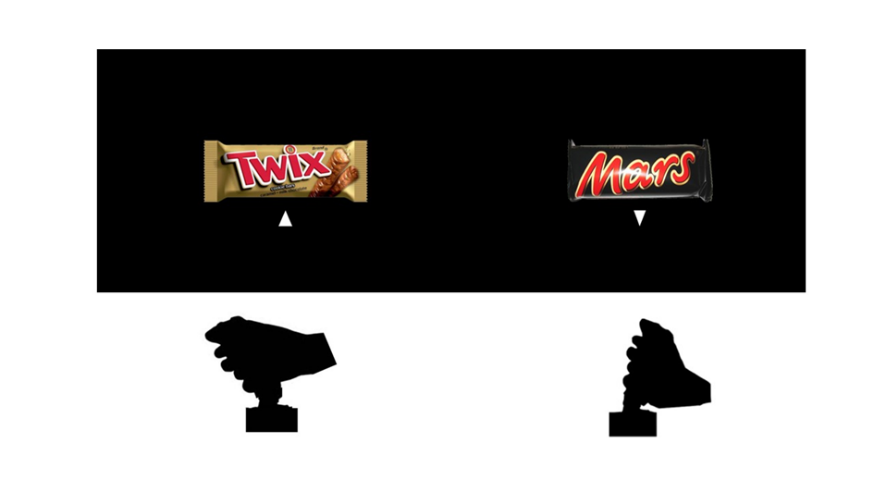


**A**

**B**

### S4. Study 1. Snack selection task

Snack selection: In person snack selection offered to participants comprising four chocolate bars and four healthier snacks (for immediate consumption):


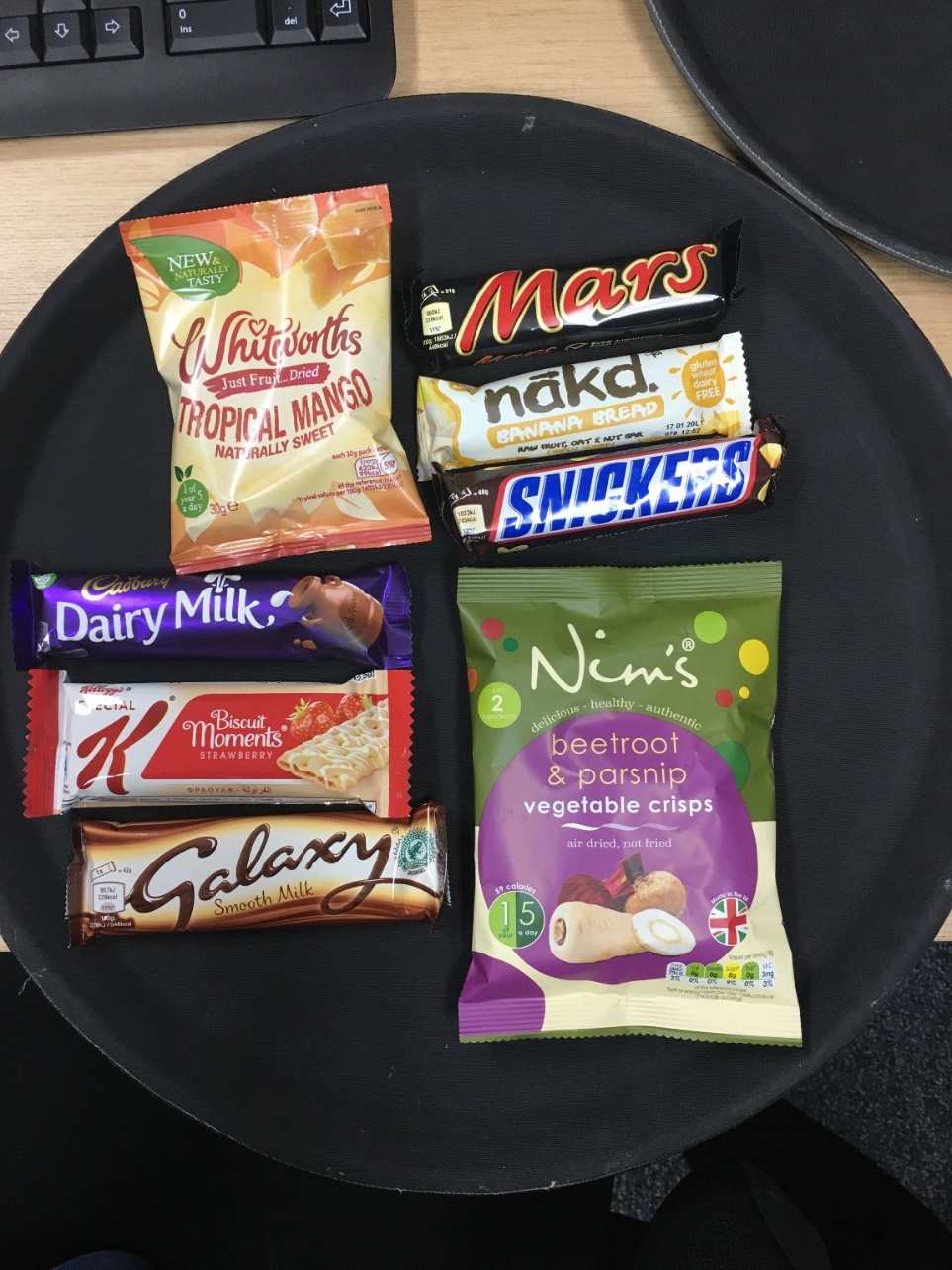


Healthier snack foods were selected based on the range of snacks used as healthier snack options in two previous studies investigating the effects of availability (Pechey et al., 2021)^[[2]](#footnote-2)^ and labelling (Clarke at al., 2020b) on selection of healthier versus less healthy snacks. Both the chocolate bars and healthier options were individually packaged single serving snacks.

### S5. Study 1. Participant characteristics by study arm

|  | | **Study arm** | | | | | |
| --- | --- | --- | --- | --- | --- | --- | --- |
|  |  | **Health Warning Label**  **(n=30)** | | **Irrelevant**  **Aversive Label**  **(n=30)** | | **No label**  **(n=30)** | |
|  |  | n* | %* | n* | %* | n* | %* |
| Gender | Male | 16 | 53 | 12 | 40 | 17 | 57 |
|  | Female | 14 | 47 | 17 | 57 | 13 | 43 |
| Age | Mean (SD) | 39.5 (13.1) | | 33.4 (12.8) | | 32.2 (10.8) | |
| Ethnicity | White background | 23 | 78 | 24 | 80 | 22 | 73 |
|  | Other | 7 | 23 | 6 | 20 | 8 | 27 |
| Highest qualification | No Bachelor degree | 13 | 43 | 15 | 50 | 15 | 50 |
|  | Bachelor’s degree or higher | 17 | 57 | 14 | 47 | 15 | 50 |
| BMI | Mean (SD) | 25.7 (4.7) | | 24.1 (5.7) | | 24.5 (4.3) | |
| Income^1^ | Up to £11,499 | 2 | 7 | 2 | 7 | 4 | 13 |
|  | £11,500 – £24,999 | 2 | 7 | 4 | 13 | 6 | 20 |
|  | £25,000 – £49,999 | 15 | 50 | 10 | 33 | 11 | 37 |
|  | £50,000 or more | 11 | 37 | 11 | 37 | 7 | 23 |
|  | Prefer not to say | 0 | 0 | 3 | 10 | 2 | 7 |
| Last time you had something to eat? | 1-2 hours ago | 16 | 53 | 15 | 50 | 15 | 50 |
|  | ≥ 3 hours ago | 14 | 46 | 15 | 50 | 15 | 50 |
| Hunger^2^ | Mean (SD) | 34.9 (28.7) | | 42.8 (25.5) | | 49.1 (22.3) | |
| Time of session | AM | 14 | 46 | 10 | 32 | 8 | 26 |
|  | PM | 16 | 54 | 20 | 66 | 22 | 73 |

* Unless otherwise stated. % rounded to whole numbers. If n/group ≠ 30, data is missing. Standard deviation (SD).

^1^ Total household income per year from all sources, before tax and other deductions.

^2^ Participant was asked “How hungry are you?” on a scale of 0 (not hungry at all) to 100 (the most hungry).

### S6. Study 1. Implicit motivation change score (in ms) between study arms (pre-conditioning implicit motivation minus post-conditioning implicit motivation) (n=87^)

|  | **Study arm** | | | | | | | | | | | |
| --- | --- | --- | --- | --- | --- | --- | --- | --- | --- | --- | --- | --- |
|  | **Health Warning Label (n=29)** | | | | **Irrelevant Warning Label (n=29)** | | | | **No Label (n=29)** | | | |
|  | Mean (95% CI) | SD | Min | Max | Mean (95% CI) | SD | Min | Max | Mean (95% CI) | SD | Min | Max |
| Implicit motivation change score | -9  (-30, 13) | 56 | -126 | 151 | 15  (-7, 36) | 57 | -69 | 178 | 2  (-14, 19) | 45 | -75 | 102 |

Standard deviation (SD). Min (Minimum). Max (Maximum).

^ Three participants had no data for their implicit motivation, one from each study arm, due to the fact that they made an error in 100% of their trials for an entire block (*i.e.* they had errors in 100% of their 48 approach and/or 48 avoid trials, in the pre-conditioning and/or post-conditioning phases).

### S7. Study 1. The number of errors in the joystick (implicit motivation) task between study arms

|  | | **Study arm** | | | | | |
| --- | --- | --- | --- | --- | --- | --- | --- |
|  |  | **Health Warning Label**  **(n = 30)** | | **Irrelevant Warning Label**  **(n = 30)** | | **No Label**  **(n = 30)** | |
|  |  | n* | %* | n* | %* | n* | %* |
| **Errors pre-conditioning** | No errors | 21 | 70 | 25 | 83 | 21 | 70 |
|  | ≥ 1 error | 9 | 30 | 5 | 17 | 9 | 30 |
| **Errors post-conditioning** | No errors | 26 | 87 | 30 | 100 | 24 | 80 |
|  | ≥ 1 error | 4 | 13 | 0 | 0 | 6 | 20 |
| **Total errors** | No errors | 17 | 57 | 25 | 83 | 20 | 67 |
|  | ≥ 1 error | 13 | 43 | 5 | 17 | 10 | 33 |
| **Mean number of errors per participant (SD) - pre-conditioning** | | 1.6 (6.4) | | 1.7 (8.6) | | 2.0 (6.6) | |
| **Mean number of errors per participant (SD) - post-conditioning** | | 1.7 (8.8) | | 0 (0) | | 1.1 (4.6) | |
| **Mean number of errors per participant (SD) - total** | | 3.3 (10.6) | | 1.7 (8.6) | | 3.1 (10.5) | |

Standard deviation (SD). * Unless otherwise stated.

### S8. Study 2. Health warning labels.

Three of the HWLs from Study 1 were used again for Study 2 (see below).


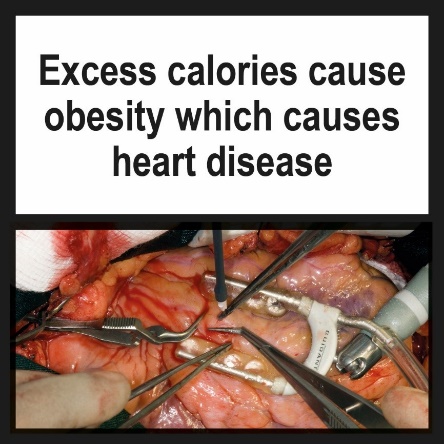

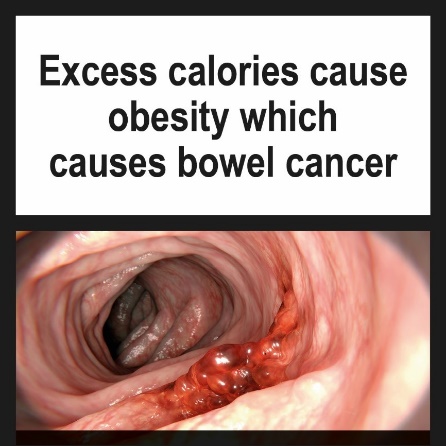

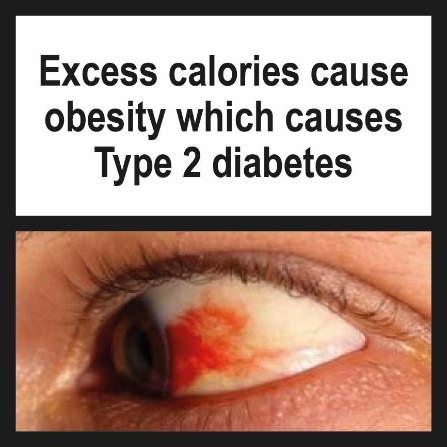


HWLs were displayed on the chocolate bar packaging, to represent what an actual labelled chocolate bar could look like while also ensuring visibility of both the label and the chocolate bar branding:


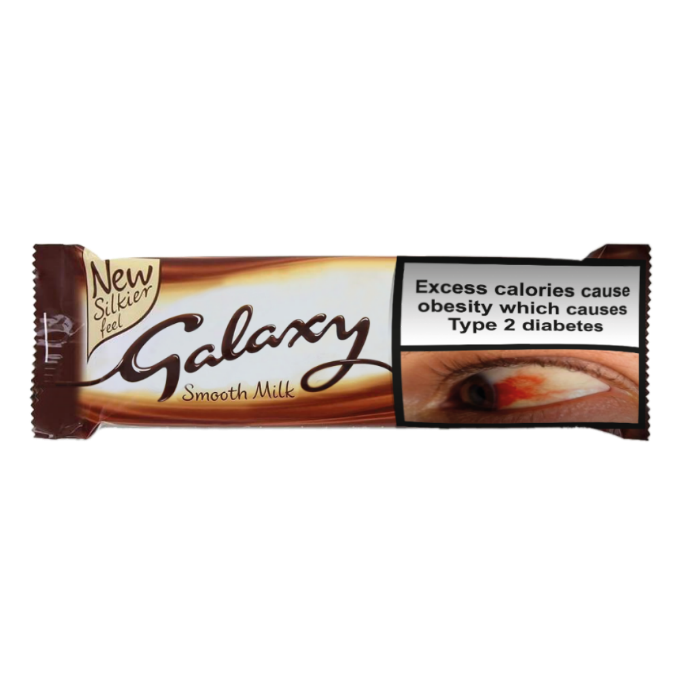

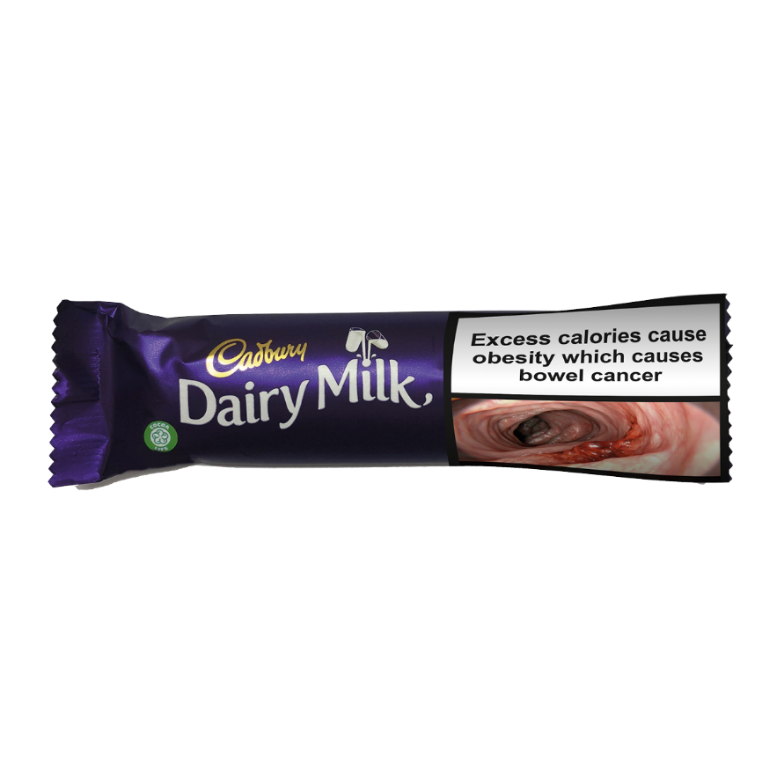

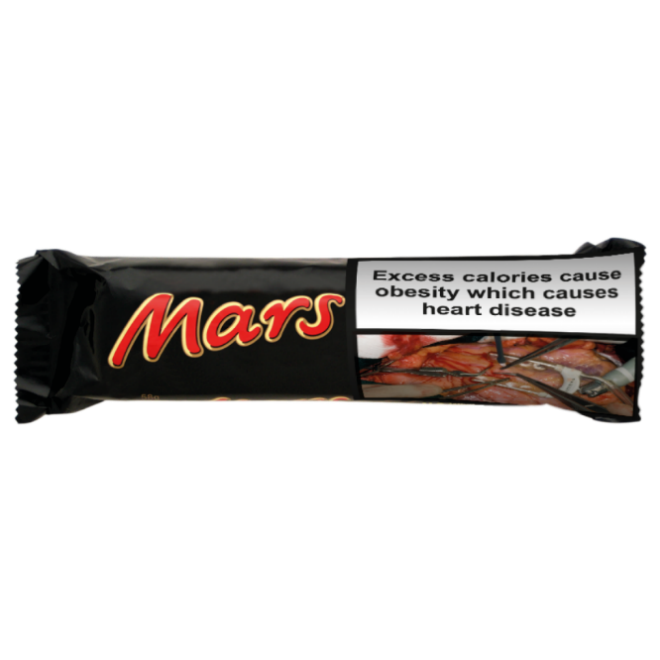


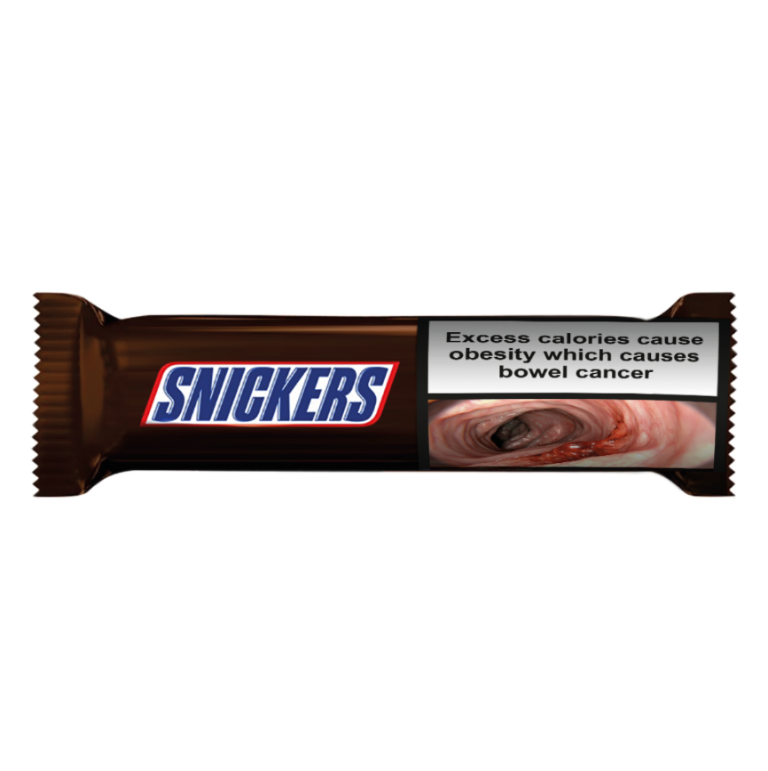


### S9. Study 2. Irrelevant aversive labels

As in Study 1, IALs were created using images from the International Affective Picture System, and formed a coherent grouping in terms of content that were distinct in being irrelevant to chocolate bar consumption, and included a text statement that explained the content of the image using a comparable amount of words to the HWLs. In Study 1, irrelevant images for the IALs were selected via an internal pilot with colleagues. To ensure the HWLs and IALs were matched on negative emotional arousal, for Study 2 we expanded on our internal pilot by running an online survey with 256 participants from the general population, who rated the three HWLs and a range of 15 potential IALs (see supplementary material for survey details). The three IALs that most closely matched in negative emotional arousal score with the three HWLs in the online survey were selected. This included one of the original IALs used in Study 1, and two new ones found to be closer in negative emotional arousal to the HWLs. See below.


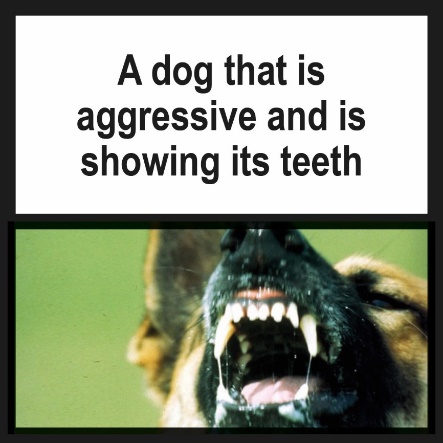

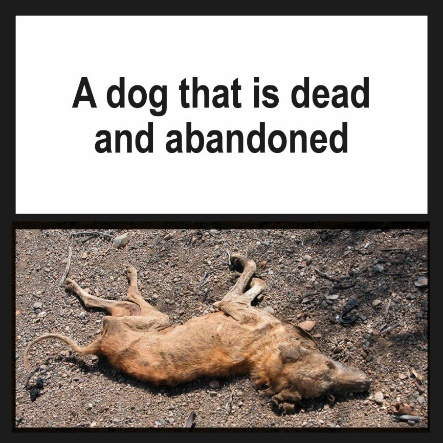

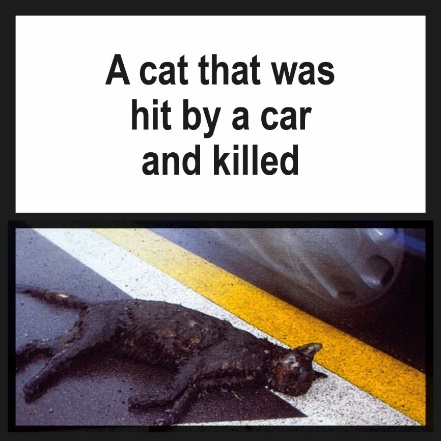


IALs were displayed on the chocolate bar packaging, to represent what an actual labelled chocolate bar could look like while also ensuring visibility of both the label and the chocolate bar branding:


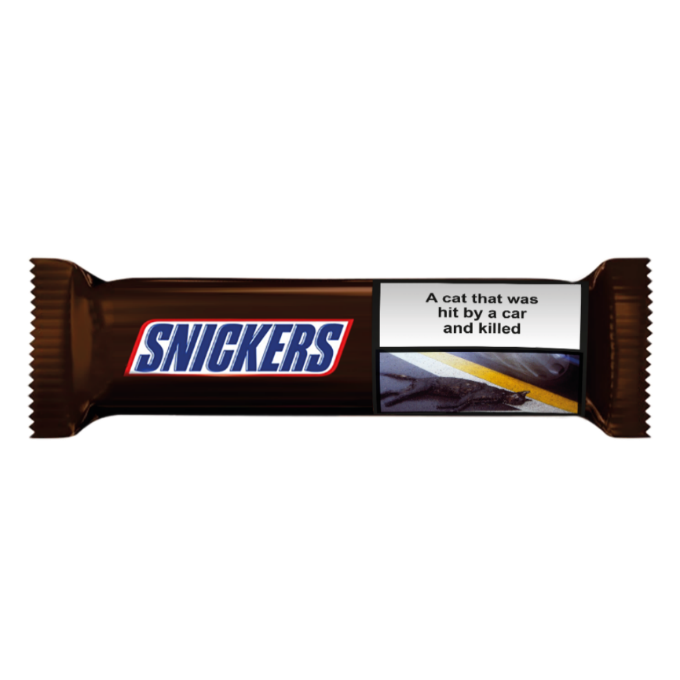

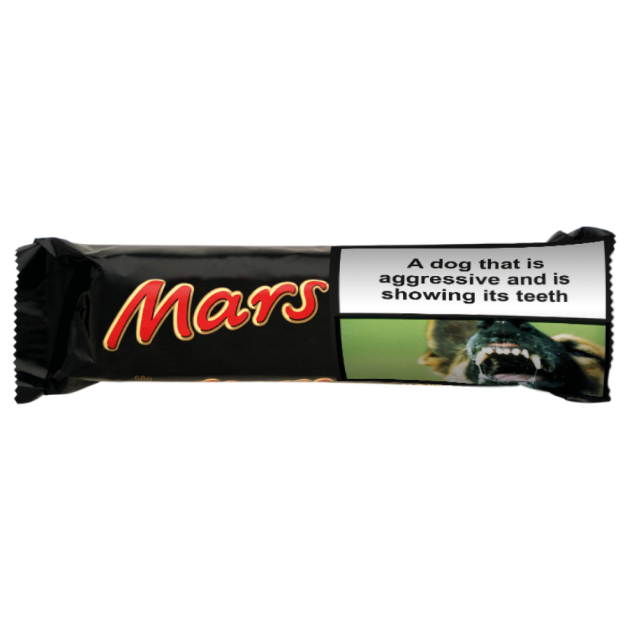

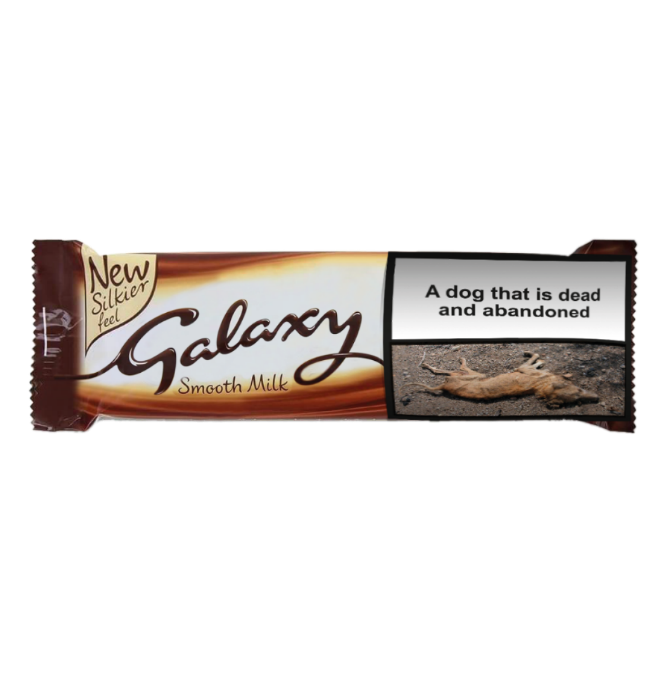

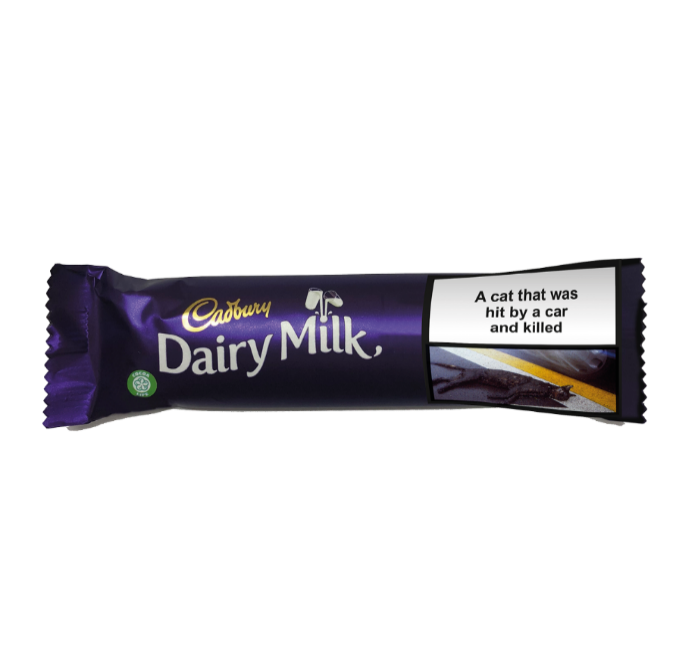


### S10**.** Study 2. **Stationery stimuli**

###
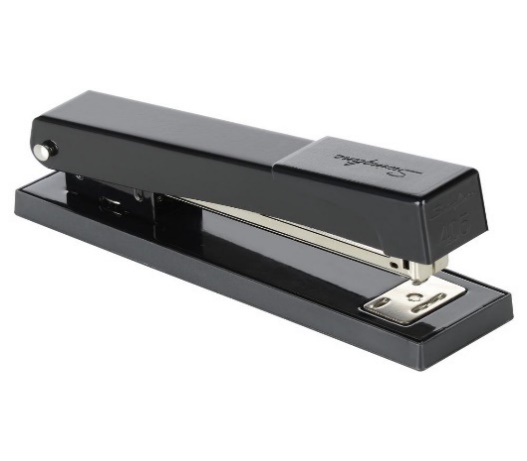


**A**

**B**


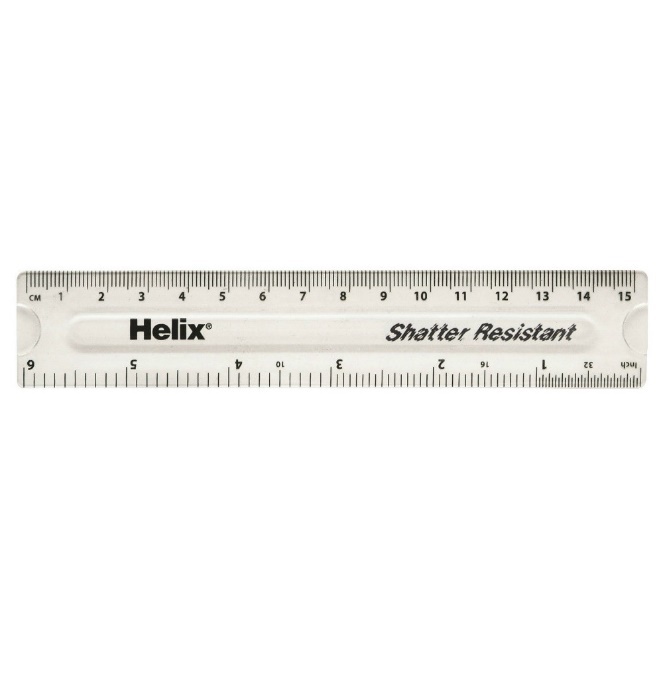


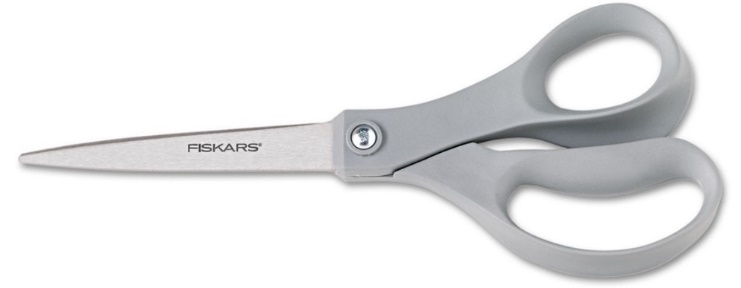


**C**

**D**


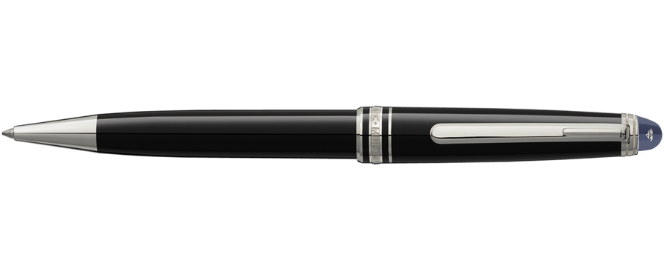


### S11. Study 2. Manikin task


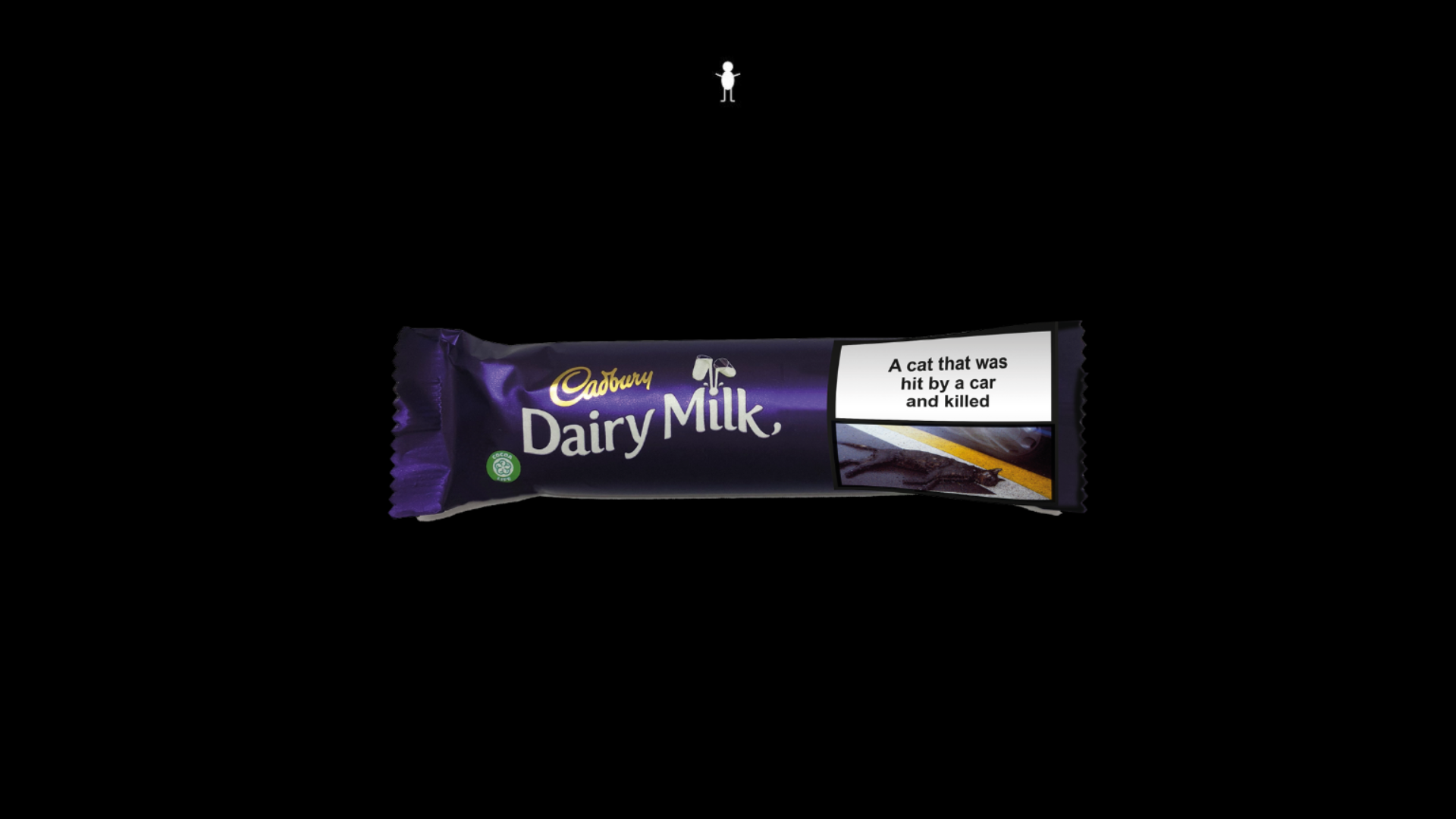


### S12. Study 2. Go/no-go task


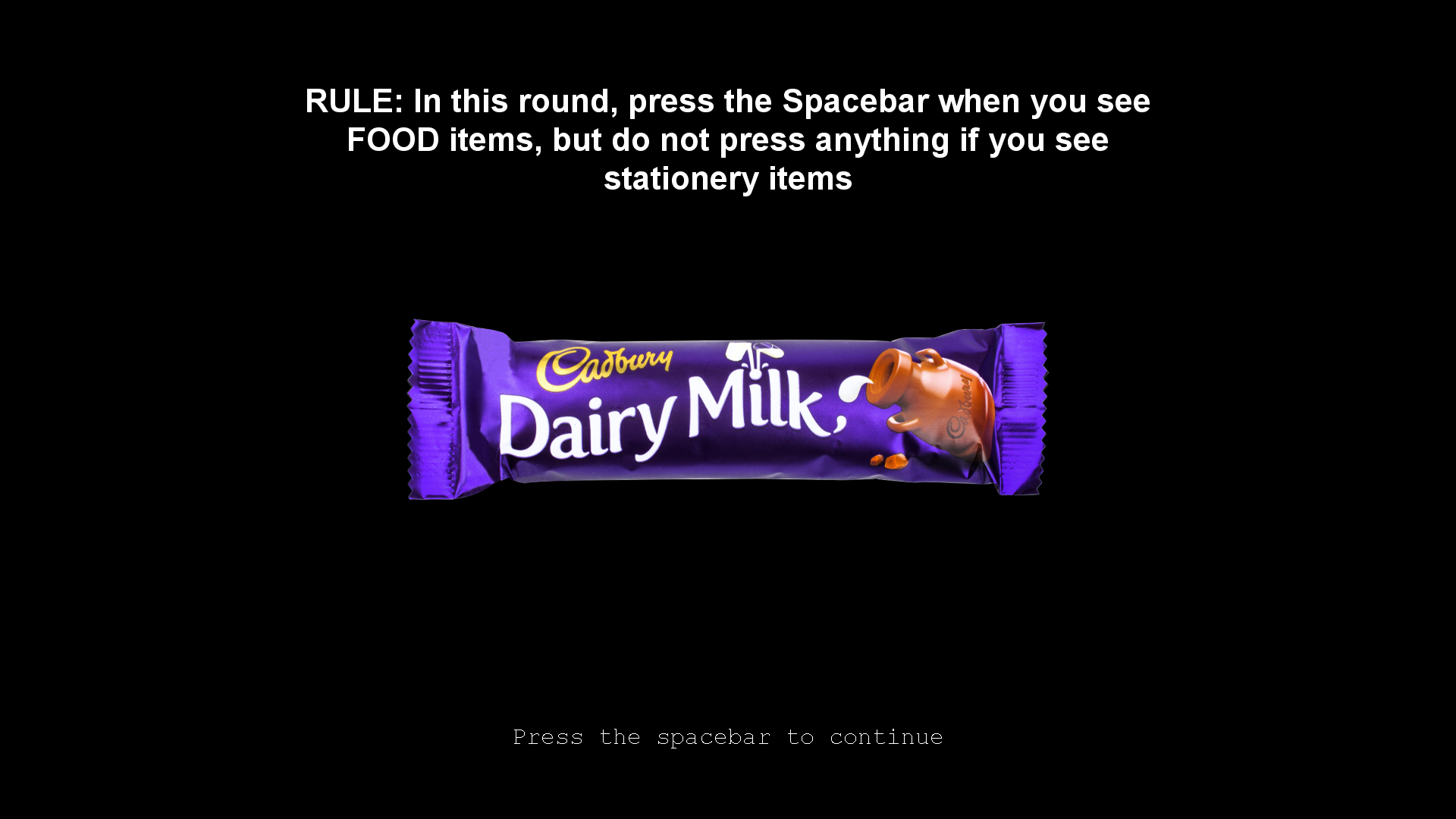


### S13. Study 2. Hypothetical snack selection tasks

### Unlabelled snack selection task:
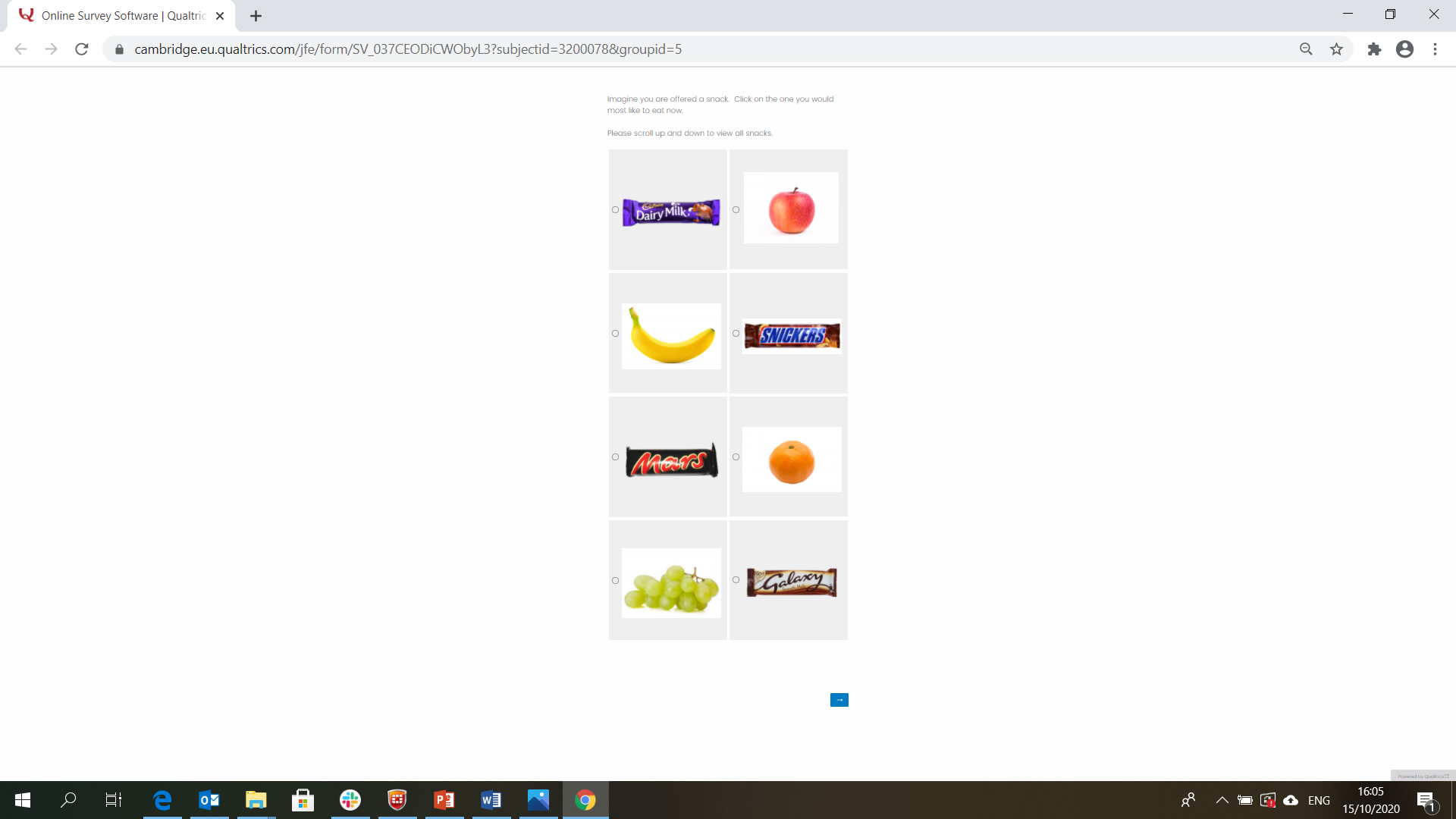


Labelled per study arm selection task (example below illustrates the selection in the HWL condition):


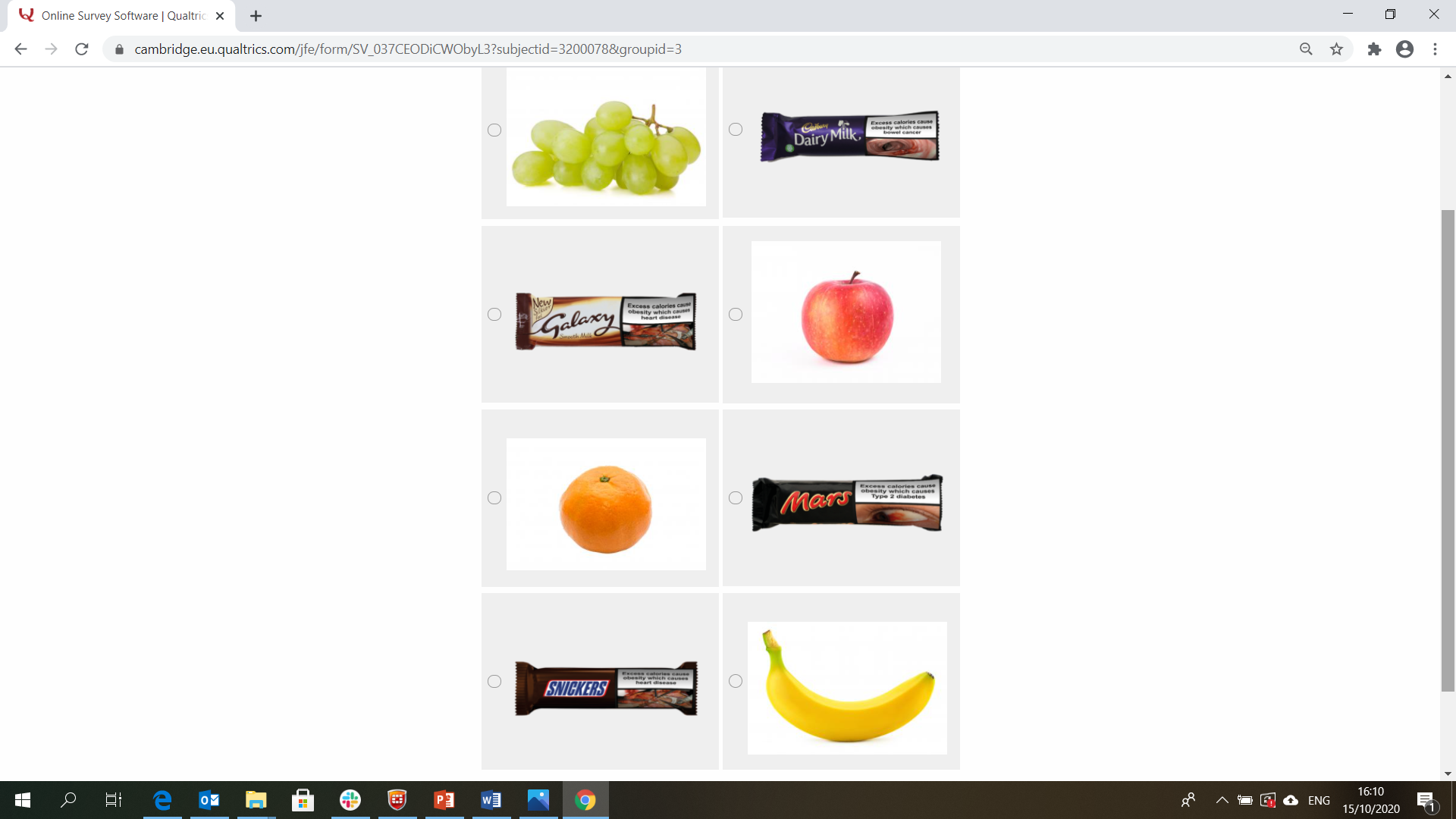


### S14. Study 2. CONSORT flow diagram

## **Analysed** (n= 1382)

Analysed (**IAL group)** (n= 454)

Excluded from the manikin task analysis (missing data) (n= 7)

Excluded from the go/no-go task analysis (missing data) (n= 0)

Analysed (**no label group**) (n= 471)

Excluded from the manikin task analysis (missing data) (n= 1)

Excluded from the go/no-go task analysis (missing data) (n= 1)

Analysed (**HWL group**) (n= 457)

Excluded from the manikin task analysis (missing data) (n= 4)

Excluded from the go/no-go task analysis (missing data) (n= 2)

Allocated to the **HWL group** (n=542)

Dropped out (n=85)

Allocated to the **IAL group** (n=533)

Dropped out (n=79)

Allocated to the **no label group** (n= 561)

Dropped out (n=90)

Drop out (most likely due to plug in download) (n= 1046)

## **Allocation**

## Randomized (n= 1636)

Eligible and sent to download plug-in required to complete manikin task (n=2682)

Ineligible (n= 2991)

- Mobile device used: 2474
- Consumption of chocolate less than weekly: 165
- Failed attention check: 271
- Incompletes: 81

## **Enrollment**

Consented to take part (n= 5673)

### S15. Study 2. Participant characteristics by study arm

|  | | **Study arm** | | | | | |
| --- | --- | --- | --- | --- | --- | --- | --- |
|  |  | **HWL (n=457)** | | **IAL (n=454)** | | **No label (n=471)** | |
|  |  | n* | %* | n* | %* | n* | %* |
| Gender | Male | 218 | 48 | 235 | 52 | 217 | 46 |
|  | Female | 237 | 52 | 218 | 48 | 253 | 54 |
|  | Other | 0 | 0 | 1 | < 1 | 1 | < 1 |
|  | Prefer not to say | 2 | < 1 | 0 | 0 | 0 | 0 |
| Age (years) | Mean (SD), range | 34 (13), 18-73 | | 34 (13), 18-76 | | 35 (12), 18-76 | |
| Ethnicity | White | 334 | 73 | 335 | 74 | 356 | 76 |
|  | Other | 120 | 26 | 116 | 26 | 114 | 24 |
|  | Prefer not to say | 3 | < 1 | 3 | < 1 | 1 | < 1 |
| Education | No Bachelor's degrees | 177 | 39 | 177 | 39 | 178 | 38 |
|  | Bachelor's degree or higher | 275 | 60 | 277 | 61 | 293 | 62 |
|  | Prefer not to say | 5 | 1 | 0 | 0 | 0 | 0 |
| Income^1^ | Up to £11,499 | 44 | 10 | 47 | 10 | 42 | 9 |
|  | £11,500 – £24,999 | 91 | 20 | 84 | 19 | 75 | 16 |
|  | £25,000 – £49,999 | 163 | 36 | 156 | 34 | 170 | 36 |
|  | £50,000 or more | 141 | 31 | 150 | 33 | 168 | 36 |
|  | Prefer not to say | 18 | 4 | 17 | 4 | 16 | 3 |
| BMI (kg/m2) | Mean (SD), range | 26 (6), 14-47 | | 26 (6) 15-51 | | 26 (6), 14-54 | |
| BMI grouped | Underweight | 23 | 5 | 19 | 4 | 16 | 3 |
|  | Healthy weight | 202 | 45 | 212 | 47 | 215 | 46 |
|  | Overweight | 121 | 27 | 129 | 27 | 134 | 29 |
|  | Obese | 71 | 16 | 52 | 12 | 59 | 13 |
|  | Severely obese | 19 | 4 | 26 | 6 | 33 | 7 |
|  | Morbidly obese | 16 | 4 | 11 | 2 | 8 | 2 |
| Hunger^2^ | Mean (SD), range | 42 (27), 0-100 | | 44 (28), 0-100 | | 41 (27), 0-100 | |
| Time since last ate | < 1 hour ago | 151 | 33 | 136 | 30 | 149 | 32 |
|  | 1 – 2 hours ago | 138 | 30 | 142 | 31 | 144 | 31 |
|  | 3 – 4 hours ago | 94 | 21 | 92 | 20 | 86 | 18 |
|  | 5 or more hours ago | 74 | 16 | 84 | 19 | 92 | 20 |
| Purchasing frequency^3^ | Dairy Milk, mean (SD) | 1.4 (0.5) | | 1.4 (0.5) | | 1.4 (0.5) | |
|  | Mars, mean (SD) | 1.1 (0.4) | | 1.2 (0.4) | | 1.2 (0.4) | |
|  | Snickers, mean (SD) | 1.2 (0.5) | | 1.2 (0.5) | | 1.2 (0.4) | |
|  | Galaxy, mean (SD) | 1.3 (0.5) | | 1.3 (0.5) | | 1.3 (0.5) | |
| Consumption frequency^4^ | Dairy Milk, mean (SD) | 1.4 (0.6) | | 1.5 (0.6) | | 1.4 (0.5) | |
|  | Mars, mean (SD) | 1.1 (0.4) | | 1.2 (0.4) | | 1.2 (0.4) | |
|  | Snickers, mean (SD) | 1.3 (0.5) | | 1.3 (0.5) | | 1.3 (0.5) | |
|  | Galaxy, mean (SD) | 1.3 (0.5) | | 1.3 (0.5) | | 1.3 (0.5) | |
| Eating behaviour^5^ | "I deliberately take small helpings to control my weight", mean (SD) | 2.3 (0.9) | | 2.2 (0.9) | | 2.3 (0.9) | |
|  | "I don't eat some foods because they make me fat.", mean (SD) | 2.3 (0.9) | | 2.3 (0.9) | | 2.3 (1.0) | |
|  | "I consciously hold back on how much I eat at meals to keep from gaining weight", mean (SD) | 2.3 (0.9) | | 2.2 (0.9) | | 2.2 (0.9) | |

*unless otherwise stated. % rounded to whole numbers. If data ≠ 100 %, data is missing.

Irrelevant aversive labels (IAL). Health warning labels (HWL). Standard deviation (SD)

^1^ Total household income per year from all sources, before tax and other deductions.

^2^ Participant was asked “How hungry are you?" on a scale of 0 (not hungry at all) to 100 (the most hungry).

^3^ Participant was asked “How many times have you purchased this chocolate bar in the last month?” (1=Monthly or less often; 2=At least once a week; 3=Just about every day)

^4^ Participant was asked “How many times have you eaten this chocolate bar in the last month?” (On a scale 1=Monthly or less often; 2=At least once a week; 3=Just about every day)

^5^ On a scale: 1= Definitely false; 2= Mostly False; 3= Mostly True; 4= Definitely True

### S16. Study 2. Exploratory variables between study arms

|  |  | **Study arm** | | |
| --- | --- | --- | --- | --- |
|  |  | **HWL (n=457)** | **IAL (m=454)** | **No label (n=471)** |
| **Manikin task – Total number or errors** | Mean (SD), n | 7.1 (9.0) | 6.9 (9.0) | 6.0 (8.0) |
|  | Median (range) | 4 (0, 56) | 4 (0, 54) | 3 (0, 58) |
| **GNG task - Rate of commission errors for stationery^1^** | Mean (SD), n | 15 (18) | 16 (20) | 17 (20) |
|  | Median (range) | 17 (0, 100) | 17 (0, 100) | 17 (0, 100) |
| **GNG task - Rate of omission errors for stationery ^1^** | Mean (SD), n | 3 (12) | 4 (16) | 3 (14) |
|  | Median (range) | 0 (0, 100) | 0 (0, 100) | 0 (0, 100) |
| **GNG task - Response time for stationery^1^** | Mean (SD), n | 467.0 (77.6) | 464.6 (93.2) | 486.7 (87.8) |
|  | Median (range) | 452 (206, 837) | 449 (315, 1303) | 473 (202, 1150) |
| **Mean liking for stationery (baseline)^1, 2^** | Mean (SD), n | 38.7 (22.5) | 40.9 (22.1) | 39.1 (23.5) |
|  | Median (range) | 38 (0, 100) | 42 (0, 100) | 39 (0, 100) |
| **Mean wanting for stationery (baseline)^1, 3^** | Mean (SD), n | 12.3 (15.8) | 13.6 (17.0) | 12.3 (17.2) |
|  | Median (range) | 5 (0, 100) | 7 (0, 100) | 5 (0, 100) |
| **Mean liking for stationery (post intervention)^1, 2^** | Mean (SD), n | 36.3 (24.4) | 40.4 (23.8) | 36.7 (24.8) |
|  | Median (range) | 35.0 (0-100) | 41.3 (0-100) | 36.3 (0-100) |
| **Mean wanting for stationery (post intervention)^1, 3^** | Mean (SD), n | 13.2 (18.0) | 15.2 (18.9) | 16.9 (20.7) |
|  | Median (range) | 5.3 (0-100) | 6.8 (0-100) | 8.8 (0-98) |

Go/no-go task (GNG). Irrelevant aversive labels (IAL). Health warning labels (HWL).

^1^ Stationery items did not have labels on them and were presented in the HWL, IAL and no label groups as control stimuli.

^2^ Explicit liking responses measured on a 100mm visual analogue scale, labelled at either end by ‘not at all’ (0), to ‘very’ (100).

^3^ Explicit wanting responses measured on a 100mm visual analogue scale, labelled at either end by ‘not at all’ (0), to ‘very’ (100).

**S17. Study 2.** **Secondary analyses – snack selection comparisons between study arms**

| **Task** | **Which snack was selected** | **Study group** | | | | | | **Chi-squared value, p value** |
| --- | --- | --- | --- | --- | --- | --- | --- | --- |
|  |  | **No label (n=471)** | | **HWL (n=457)** | | **IAL (n=454)** | |  |
|  |  | n | **%**  **(95% CI)** | n | **%**  **(95% CI)** | n | **%**  **(95% CI)** |  |
| Snack selection 1 -**unlabelled** | Chocolate bar | 297 | 63 | 222 | 49 | 276 | 61 | χ^2^ (2) = 22.9,  < 0.001 |
|  | Healthier snack | 174 | **37**  **(33, 41)** | 235 | **51**  **(47, 56)** | 178 | **39**  **(35, 44)** |  |
| Snack selection 1 - **labelled** | Chocolate bar | 294 | 62 | 149 | 33 | 190 | 42 | χ^2^ (2) = 87.3,  < 0.001 |
|  | Healthier snack | 177 | **38**  **(33, 42)** | 308 | **67**  **(63, 72)** | 264 | **58**  **(54, 63)** |  |
| Snack selection 2 -**unlabelled** | Chocolate bar | 290 | 62 | 230 | 50 | 291 | 64 | χ^2^ (2) = 20.3, < 0.001 |
|  | Healthier snack | 181 | **38**  **(34, 43)** | 227 | **50**  **(45, 54)** | 163 | **36**  **(32, 40)** |  |
| Snack selection 2 - **labelled** | Chocolate bar | 288 | 61 | 148 | 32 | 185 | 41 | χ^2^ (2) = 82.3, < 0.001 |
|  | Healthier snack | 183 | **39**  **(35, 43)** | 309 | **68**  **(63, 72)** | 269 | **59**  **(55, 64)** |  |

Irrelevant aversive labels (IAL). Health warning labels (HWL).

**S18a. Study 2. Secondary analyses – healthy snack selection comparisons between HWL and IAL groups.**

| **Healthy snack selection** | **Healthy snack selection** | | | | |
| --- | --- | --- | --- | --- | --- |
|  | **HWL (n=457)** | | **IAL (n=454)** | | **Chi-squared value, p value** |
|  | n | **%**  **(95% CI)** | n | **%**  **(95% CI)** |  |
| Snack selection 1 -**unlabelled** | 235 | **51**  **(47, 56)** | 178 | **39**  **(35, 44)** | χ^2^ (1) = 13.713, p < 0.001 |
| Snack selection 1 - **labelled** | 308 | **67**  **(63, 72)** | 264 | **58**  **(54, 63)** | χ^2^ (1) = 8.334, p = 0.004 |
|  |  |  |  |  |  |
| Snack selection 2 -**unlabelled** | 227 | **50**  **(45, 54)** | 163 | **36**  **(32, 40)** | χ^2^ (1) = 17.635, p < 0.001 |
| Snack selection 2 - **labelled** | 309 | **68**  **(63, 72)** | 269 | **59**  **(55, 64)** | χ^2^ (1) = 6.869, p = 0.009 |

Irrelevant aversive labels (IAL). Health warning labels (HWL).

**S18b. Study 2. Secondary analyses – healthy snack selection comparisons between labelled and unlabelled**

|  | **Healthy snack selection** | | | | | | | | |
| --- | --- | --- | --- | --- | --- | --- | --- | --- | --- |
|  | **No label (n=471)** | | **Z, p value*** | **HWL (n=457)** | | **Z, p value*** | **IAL (n=454)** | | **Z, p value*** |
|  | n | **%**  **(95% CI)** |  | n | **%**  **(95% CI)** |  | n | **%**  **(95% CI)** |  |
| Snack selection 1 -**unlabelled** | 174 | **37**  **(33, 41)** | 0.46,  0.694 | 235 | **51**  **(47, 56)** | 6.97,  < 0.001 | 178 | **39**  **(35, 44)** | 8.28,  < 0.001 |
| Snack selection 1 - **labelled** | 177 | **38**  **(33, 42)** |  | 308 | **67**  **(63, 72)** |  | 264 | **58**  **(54, 63)** |  |
|  |  |  |  |  |  |  |  |  |  |
| Snack selection 2 -**unlabelled** | 181 | **38**  **(34, 43)** | 0.46,  0.694 | 227 | **50**  **(45, 54)** | 7.84,  < 0.001 | 163 | **36**  **(32, 40)** | 10.55,  < 0.001 |
| Snack selection 2 - **labelled** | 183 | **39**  **(35, 43)** |  | 309 | **68**  **(63, 72)** |  | 269 | **59**  **(55, 64)** |  |

Irrelevant aversive labels (IAL). Health warning labels (HWL).

*p values were calculated using the 95% CI of the proportion

1. Pechey, E., Clarke, N., Mantzari, E., Blackwell, A. K., De-Loyde, K., Morris, R. W., Marteau, T.M., & Hollands, G. J. (2020). Image-and-text health warning labels on alcohol and food: potential effectiveness and acceptability. BMC Public Health, 20, Article 376. [↑](#footnote-ref-1)
2. Pechey, R., Sexton, O., Codling, S., & Marteau, T.M. (2021). Impact of increasing the availability of healthier vs. less-healthy food on food selection: A randomised laboratory experiment. BMC Public Health, 21, 132. [↑](#footnote-ref-2)
